# Unusual Polyhydroxylated Steroids from the Starfish *Anthenoides laevigatus*, Collected off the Coastal Waters of Vietnam

**DOI:** 10.3390/molecules25061440

**Published:** 2020-03-23

**Authors:** Alla A. Kicha, Dinh T. Ha, Timofey V. Malyarenko, Anatoly I. Kalinovsky, Roman S. Popov, Olesya S. Malyarenko, Tran T. T. Thuy, Pham Q. Long, Nguyen T. T. Ha, Natalia V. Ivanchina

**Affiliations:** 1 G.B. Elyakov Pacific Institute of Bioorganic Chemistry, Far Eastern Branch of Russian Academy of Sciences, Pr. 100-let Vladivostoku 159, 690022 Vladivostok, Russia; malyarenko-tv@mail.ru (T.V.M.); kaaniw@piboc.dvo.ru (A.I.K.); prs_90@mail.ru (R.S.P.); malyarenko.os@gmail.com (O.S.M.); ivanchina@piboc.dvo.ru (N.V.I.); 2 Institute of Natural Products Chemistry, Vietnam Academy of Science and Technology, 18 Hoang Quoc Viet, Cau Giay, Hanoi, Vietnam; dinhha.inpc@gmail.com; 3 Graduate University of Science and Technology, Vietnam Academy of Science and Technology, 18 Hoang Quoc Viet, Cau Giay, Hanoi, Vietnam; mar.biochem@fpt.vn; 4 School of Natural Sciences, Far Eastern Federal University, Sukhanova Str. 8, 690000 Vladivostok, Russia; 5 Institute of Chemistry, Vietnam Academy of Science and Technology, 18 Hoang Quoc Viet, Cau Giay, Hanoi, Vietnam; thuha.vast@gmail.com

**Keywords:** polyhydroxylated steroids, NMR spectra, starfish, *Anthenoides laevigatus*, cytotoxicity, soft agar assay

## Abstract

Four new polyhydroxylated steroids **1**–**4** were isolated along with two previously known related steroids **5** and **6** from the methanolic extract of the starfish *Anthenoides laevigatus* collected off the coastal waters of Vietnam. Structures of new compounds were substantially elucidated by one-dimensional (1D) and two-dimensional (2D) NMR spectroscopy and HRESIMS techniques. Heptaol **1** and hexaol **2** contain the common 5α-cholestane skeleton, while hexaol **3** and heptaol **4** have the rare among starfish steroid compounds 5β-cholestane skeleton. Compounds **1**, **5**, and **6** do not show cytotoxic effects against normal JB6 Cl41 and cancer HT-29 and MDA-MB-231 cells, however they inhibit cell proliferation and colony formation of cancer HT-29 and MDA-MB-231 cells.

## 1. Introduction

Polyhydroxylated steroids have been found in diverse marine species of ophiuroids, gorgonians, sponges, and other marine invertebrates. However, the class Asteroidea (also known as starfish or sea stars) is the richest source of these kind of steroids [1,2,3,4,5,6,7]. These substances generally contain from four to nine hydroxyl groups in the steroid nucleus and side chains and are characterized by a wide variety of chemical structures. Polyhydroxylated steroids are present in starfish in both free, sulfated, or glycosylated by one to three monosaccharide residues forms. These compounds usually occur in very complicated mixtures that are often difficult to separate into individual components by chromatographic methods. In addition to the original chemical structure, polyhydroxylated steroids attract attention with a wide diversity of biological effects including neuritogenic, neuroprotective, antiviral, anti-inflammatory, immunomodulatory, and other activities [7,8]. Recently, new knowledge about the cancer preventive activity and toxicity against different human tumor cell lines and molecular mechanisms of action of some starfish steroid compounds has been acquired [9]. Moreover, for the first time, it has been shown that starfish polyhydroxylated compounds demonstrated the radio sensitizing activity that was realized through apoptosis induction by the regulation of anti- and pro-apoptotic protein expression followed by activation of caspases and DNA degradation [10]. On the basis of the data obtained, we assume that the study of polyhydroxylated steroids from the starfish could lead to new promising results on their biological activities.

The search for new metabolites from starfish is a long-term direction of the studies of G.B. Elyakov Pacific Institute of Bioorganic Chemistry which celebrated its 55th anniversary in 2019. Continuing our research on biologically active steroid metabolites from the starfish species inhabiting the Vietnamese sea waters [11,12,13,14,15], herein, we describe the results of our investigation of steroid constituents from the methanolic extract of the starfish *Anthenoides laevigatus* Liao & A.M. Clark, 1989 (order Valvatida, family Goniasteridae), collected off the coastal waters of the Qui Nhon Province, Vietnam. We have isolated and structurally studied four new polyhydroxylated steroids **1**−**4,** along with two previously known related steroids **5** and **6**. Additionally, the action of compounds **1**, **5**, and **6** on cell viability and proliferation of normal and cancer cells, as well as colony formation of cancer cells in a soft agar clonogenic assay *in vitro*, have been investigated.

## 2. Results and Discussion

### 2.1. Structure Elucidation of Compounds ***1***−***6***

**Figure 1:**
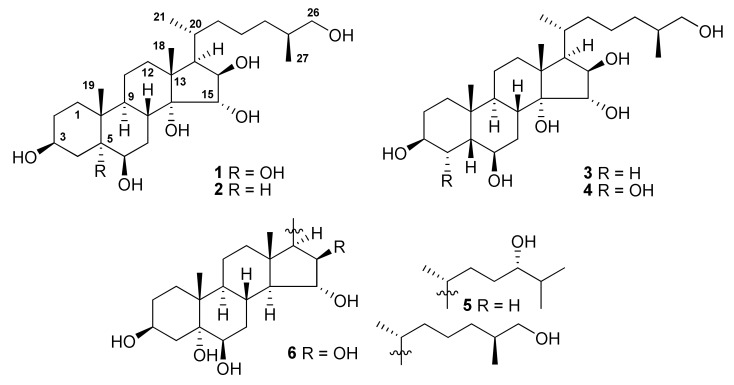
Structures of compounds **1**−**6** isolated from *A. laevigatus.*

The concentrated methanol extract of *A. laevigatus* was subjected to sequential separation by column chromatography using Amberlite XAD-2 silica gel followed by reversed-phase HPLC on Discovery C18 and YMC-Pack Pro C18 columns to yield four new polyhydroxylated steroids (**1**−**4**), along with two known compounds **5** and **6** (Figure 1). The known polyhydroxylated steroids were identified by analysis of their ^1^H-, ^13^C-NMR, and ESIMS spectra and comparison with those reported earlier for (24*S*)-5*α*-cholestane-3*β*,5,6*β*,15*α*,24-pentaol (**5**) and related sulfated compounds previously isolated from the starfish *Luidia clathrata* and *Henricia leviuscula* [16,17], and (25*S*)-5*α*-cholestane-3*β*,5,6*β*,15*α*,16*β*,26-hexaol (**6**) from the starfish *L. clathrata* [18].

**Figure 2:**
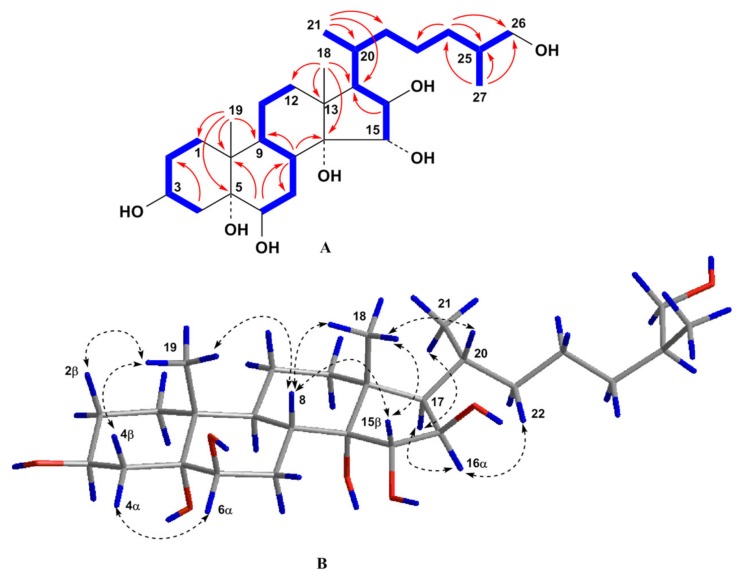
(**A**) COSY and key HMBC correlations for compound **1**; (**B**) Key ROESY correlations for compound **1**. Colors reveal the atoms of hydrogen (blue), oxygen (red) and carbon (grey) and their bonds.

The molecular formula of compound **1** was established to be of C_27_H_48_O_7_ from the [M + Na]^+^ sodium adduct ion peak at *m*/*z* 507.3292 in the (+)HRESIMS spectrum and the [M – H]^–^ deprotonated ion peak at *m*/*z* 483.3321 in the (–)HRESIMS spectrum (Figure S1). The ^1^H- and ^13^C-NMR spectroscopic data of **1** displayed the proton and carbon chemical shifts of two angular methyl groups CH_3_-18 (*δ*_H_ 1.00 s and *δ*_C_ 16.5) and CH_3_-19 (*δ*_H_ 1.17 s and *δ*_C_ 17.3), one oxygenated methylene CH_2_-26 (*δ*_H_ 3.42 dd (*J* = 10.5, 5.7), 3.30 m and *δ*_C_ 68.4), four oxygenated methines CH-3 (*δ*_H_ 4.00 m and *δ*_C_ 68.3), CH-6 (*δ*_H_ 3.49 t (*J* = 3.0) and *δ*_C_ 76.5), CH-15 (*δ*_H_ 3.86 d (*J* = 3.3) and *δ*_C_ 85.6), and CH-16 (*δ*_H_ 3.96 dd (*J* = 8.1, 3.3) and *δ*_C_ 82.1), and two oxygenated tertiary carbons C-5 (*δ*_C_ 76.6) and C-14 (*δ*_C_ 82.7) (Table 1 and Table 2, Figures S2 and S3). These signals were similar to the corresponding resonances in the NMR spectra of co-occuring compound **6,** except for the proton and carbon resonances of C-14 and CH-15. Therefore, the doublet of doublets of H-15 at *δ*_H_ 3.73 (*J* = 10.4, 2.4) in the ^1^H-NMR spectrum of **6** became doublet at *δ*_H_ 3.86 d (*J* = 3.3) in the ^1^H-NMR spectrum of **1** suggesting the presence of an additional hydroxyl group at C-14 in the steroid nucleus of **1** as compared with **6**. In addition, the value of the *J*_16,17_ = 8.1 Hz and the existence of the ROESY cross-peak H-16/H-17 indicated the α-orientation of the proton H-16 and, accordingly, 3β,5,6β,14,15α,16β,26-heptahydroxy substitution in **1**. An analysis of the COSY, HSQC, HMBC, and ROESY spectra ascertained all the proton and carbon signals in **1** (Table 1 and Table 2, Figures S4). The COSY and HSQC experiments led to the assignment of the proton atom sequences at C-1 to C-4, C-6 to C-12 through C-11, C-15 to C-17, C-17 to C-21 through C-20, C-20 to the end of the side chain. The key HMBC correlations H-4/C-2; H-6/C-8, C-10; H-8/C-7, C-9, C-14; H-16/C-17; H_3_-18/C-12, C-13, C-14, C-17; H_3_-19/C-1, C-5, C-9, C-10; H_3_-21/C-17, C-20, C-22; H_2_-24/C-23, C-25, C-26; and H_3_-27/C-24, C-25, C-26 confirmed the total structure of the molecule of **1** (Figure 2). The signal shape and coupling constants of protons H-3, H-6, H-15, and H-16 and the presence of the key ROESY cross-peaks Hα-4/H-6; H-8/H-15; H-16/H-17; H-17/H_3_-21; H_3_-18/H-8, H-15; and H_3_-19/Hβ-2, Hβ-4, H-8 confirmed the 3β,6β,15α,16β relative configurations of the oxygenated carbons and common 5*α*-cholestane skeleton in **1** (Figure 2). The 20*R-*configuration was assigned based on the ROESY correlations of H_3_-18/H-20 and H-16/H-22 and the downfield chemical shift of H_3_-21 at *δ*_H_ 0.90 [19]. The absolute configuration of the asymmetric center C-25 was defined by examination of ^1^H-NMR spectra of (*R*)- and (*S*)-MTPA derivatives obtained by reaction of **1** with *S*-(+)- and *R*-(−)-MTPA chlorides, respectively. The ^1^H-NMR spectrum of 3,15,26-tri**-**(*R*)-MTPA ester of **1** showed H_2_-26 signals as two close double doublets at *δ*_H_ 4.15 and 4.18, while that of 3,6,15,26-tetra-(*S*)-MTPA ester of **1** displayed two well-separated double doublets at *δ*_H_ 4.08 and 4.24 (Figure S8). These values were comparable with those of (*R*)- and (*S*)-MTPA derivatives obtained from other (25*S*)-26-hydroxy steroids [20]. Thus, the structure of compound **1** was determined to be the (25*S*)-5α-cholestane-3β,5,6β,14,15α,16β,26-heptaol.

The molecular formula of compound **2** was established to be of C_27_H_48_O_6_ from the [M + Na]^+^ sodium adduct ion peak at *m*/*z* 491.3337 in the (+)HRESIMS spectrum and the [M –H]^–^ deprotonated ion peak at *m*/*z* 467.3379 in the (–)HRESIMS spectrum (Figure S9). Along with mass-spectra, the ^1^H-, ^13^C-, and DEPT NMR spectra revealed the presence of a hexahydroxy substitution in **2**. Similarity of the corresponding proton and carbon signals, as well as coupling constants in the NMR spectra of **2** and **1,** indicated that compound **2** has the same 14,15α,16β-trihydroxy substitution in the steroid C/D rings and 26-hydroxy cholestane side chain (Table 1 and Table 2, Figures S10 and S11).

However, most of the proton and carbon chemical shifts of steroid A/B rings in the NMR spectra of **2** were quite different from those of **1**. The characteristic proton and carbon resonances of angular methyl group CH_3_-19 (*δ*_H_ 1.04 s and *δ*_C_ 16.1), an oxygenated methine CH-3 (*δ*_H_ 3.53 m and *δ*_C_ 72.4), and an oxygenated methine CH-6 (*δ*_H_ 3.77 q (*J* = 2.6) and *δ*_C_ 72.8) observed in the NMR spectra of **2** testified to the 3β,6β-dihydroxy substitution in 5α-cholestane nucleus in **2** [21]. The COSY and HSQC spectroscopic data ascertained the proton sequences at C-1 to C-8, C-8 to C-12 through C-11, C-15 to C-17, C-17 to C-21 through C-20, and C-20 to the end of the side chain (Figures S12 and S13). The key HMBC cross-peaks H-6/C-8, C-10; H-16/C-13, C-15; H_3_-18/C-12, C-13, C-14, C-17; H_3_-19/C-1, C-5, C-9, C-10 and the key ROESY cross-peaks Hα-4/H-6; H-5/H-3, Hα-7, H-9; H-16/H-17, H-22; H-17/H_3_-21; H_3_-18/H-8, H-11β, H-15, H-20; and H_3_-19/Hβ-1, Hβ-2, Hβ-4, H-8 confirmed the 3β,6β,15α,16β-tetrahydroxy pattern in 5α/9α/10β/13β steroid nucleus in **2** (Figures S14 and S15). The configuration at C-25 was determined as (*S*) by analogy with co-occurring compound **1** and similarity of the proton and carbon chemical shifts of the both side chains in the NMR spectra. Therefore, the structure of steroid **2** was established as the (25*S*)-5α-cholestane-3β,6β,14,15α,16β,26-hexaol.

**Figure 3:**
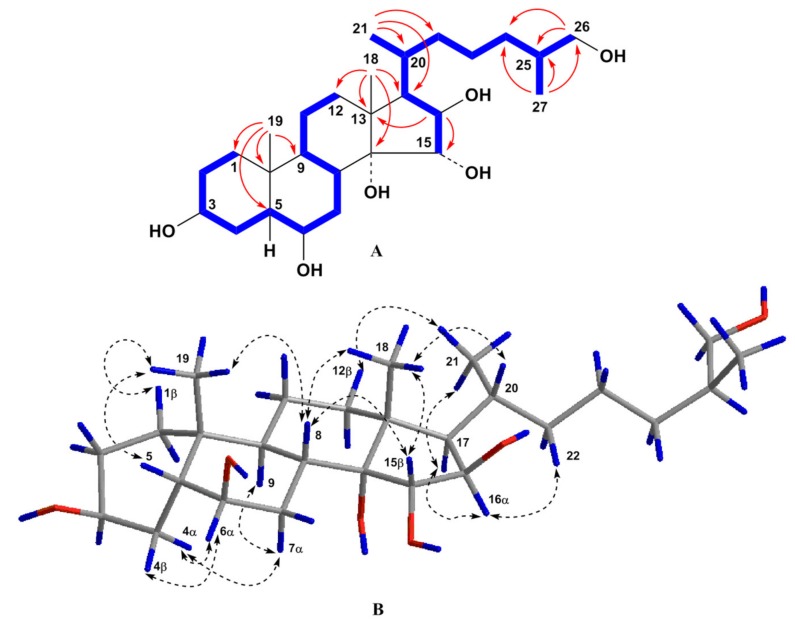
(**A**) COSY and key HMBC correlations for compound **3**; (**B**) Key ROESY correlations for compound **3**. Colors reveal the atoms of hydrogen (blue), oxygen (red) and carbon (grey) and their bonds.

According to the presence of the [M + Na]^+^ sodium adduct ion peak at *m*/*z* 491.3341 in the (+)HRESIMS spectrum and the [M – H]^–^ deprotonated ion peak at *m*/*z* 467.3380 in the (–)HRESIMS spectrum, the molecular formula C_27_H_48_O_6_ of compound **3** has been found to be identical to that of **2** (Figure S16). The detailed comparison of the ^1^H- and ^13^C-NMR spectra of compounds **3** and **2** has revealed that the proton and carbon resonances belonging to the steroid C/D rings and side chain of **3** are close to those of **2** indicating the 14,15α,16β,26-tetrahydroxy substitution in **3**, while the proton and carbon signals of the steroid A/B rings of **3** substantially differed from those of **2** (Table 1 and Table 2, Figures S17 and S18). The proton and carbon signals in the NMR spectroscopic data attributable to the A/B rings of **3** showed the presence of angular methyl group CH_3_-19 (*δ*_H_ 1.13 s and *δ*_C_ 26.3) and two oxygenated methines CH-3 (*δ*_H_ 3.99 br. q (*J* = 2.7) and *δ*_C_ 67.1) and CH-6 (*δ*_H_ 3.66 q (*J* = 2.7) and *δ*_C_ 74.3). The signal of CH_3_-19 in the ^13^C-NMR spectrum of **3** was shifted from *δ*_C_ 16.1 to 26.3 in comparison with that of **2**. This fact strongly testified to *cis*-A/B ring fusion in **3** [16,20]. The coupling constant *J* = 2.7 of the broad quartet of H-3 corresponded well to the 3β-hydroxyl group in 5β-cholestane nucleus, and the coupling constant *J* = 2.7 of the quartet of H-6 indicated the 6β-hydroxyl group in **3** [16]. All the proton and carbon signals associated with the steroid nucleus and side chain were assigned by 2D experiments (Table 1 and Table 2, Figure 3, Figures S19). Proton and carbon chemical shifts of the steroid A/B rings of **3** were similar to the corresponding data of (25*S*)-5β-cholestane-3β,6β,15α,16β,26-pentaol isolated from the starfish *L. clathrata* [16]. The key ROESY cross-peaks H_3_-19/Hβ-1, H-5, H-8; H_3_-18/H-8, H-15, H-20; Hα-4/H-6, Hα-7; Hβ-4/H-6; H-8/H-15; H-16/H-17; and H-17/H_3_-21 and proton coupling constants confirmed the 3β,6β,15α,16β relative configurations of the oxygenated carbons and the 5β-cholestane skeleton of **3** (Figure 3). As a result, steroid **3** was proved to be the *cis*-A/B ring fusion isomer of steroid **2** and its structure was established as (25*S*)-5β-cholestane-3β,6β,14,15α,16β,26-hexaol.

The molecular formula of compound **4** was established to be of C_27_H_48_O_7_ from the [M + Na]^+^ sodium adduct ion peak at *m*/*z* 507.3290 in the (+)HRESIMS spectrum and the [M – H]^–^ deprotonated ion peak at *m*/*z* 483.3327 in the (–)HRESIMS spectrum (Figure S23). Data of the mass-spectra and the ^1^H- and ^13^C-NMR spectra showed the presence of seven hydroxyl groups in **4**. The examination of the ^1^H-, ^13^C-, and 2D NMR spectra of steroids **4** and **3** revealed that both compounds have the identical 14,15α,16β-trihydroxy substitution and 26-hydroxy cholestane side chain, but the proton and carbon resonances of the steroid A/B rings of **4** differed from those of **3** (Table 1 and Table 2, Figures S24). The deshielded shift of the signal of CH_3_-19 at *δ*_C_ 25.6 in the ^13^C-NMR spectrum and the existence of the ROESY cross-peak H_3_-19/H-5 immediately showed a 5β-cholestane skeleton in **4**. The proton connectivities from C-1 to C-9 in A/B rings were ascertained using the COSY and HSQC experiments. The ^1^H- and ^13^C-NMR spectroscopic data, referred to the steroid A/B rings of **4**, revealed the proton and carbon chemical shifts of three oxygenated methines, including CH-3 (*δ*_H_ 3.70 br. q (*J* = 3.7) and *δ*_C_ 72.2), CH-4 (*δ*_H_ 3.66 t (*J* = 3.7) and *δ*_C_ 75.3), and CH-6 (*δ*_H_ 4.00 q (*J* =3.3) and *δ*_C_ 73.3). The irradiation of the proton H-5 in the 1D TOCSY experiment gave an enhancing signal of the neighboring proton H-4, that confirmed the presence of an additional hydroxyl group at C-4 in **4** as compared with **3** (Figure S30). Small values of the coupling constants of the protons H-3, H-4, and H-6 showed the absence of their axially axial interaction with neighboring protons. As a result, the 3β,4α,6β-trihydroxy pattern and the *cis*-A/B ring fusion were determined. Accordingly, the structure of **4** was established as (25*S*)-5β-cholestane-3β,4α,6β,14,15α,16β,26-heptaol.

Compounds **3** and **4** have the 5β-cholestane skeleton, which are rare among starfish steroids. Previously, only two steroid compounds with the *cis*-A/B ring junction, (25*S*)-5β-cholestane-3β,6β,15α,16β,26-pentaol from the starfish *L. clathrata* [17] and (25*S*)-5β-cholestane-3α,6β,15α,16β,26-pentaol from the starfish *Tremaster novaecaledoniae* [20], were found.

### 2.2. Biological Evaluation

#### 2.2.1. The Effect of Compounds **1**, **5**, and **6** on Cancer Cells’ Viability and Proliferation of Normal and Cancer Cells

In the first step of bioactivity investigations, the cytotoxicity of compounds **1**, **5**, and **6** was determined by measuring the metabolic activity of normal mouse epidermal JB6 Cl41, human colorectal carcinoma HT-29, and breast cancer MDA-MB-231 cells using MTS reagent. None of the tested compounds inhibited the viability of JB6 Cl41, HT-29, and MDA-MB-231 cells by 50% at concentrations up to 100 µM. The compounds **1**, **5**, and **6** decreased the cell viability by less than 20% at 100 µM (data not shown).

**Figure 4:**
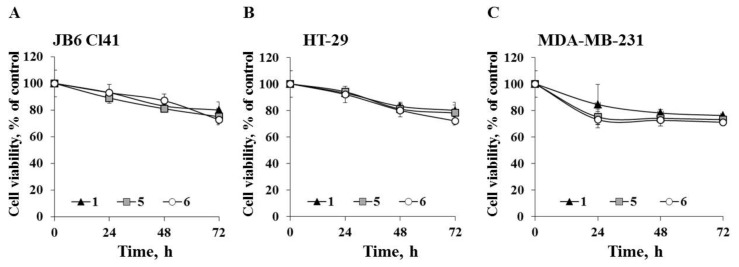
The effect of compounds **1**, **5**, and **6** on cell proliferation**.** (**A**) JB6 Cl41; (**B**) HT-29; or (**C**) MDA-MB-231 cells were treated with compounds **1**, **5**, and **6** at concentration of 20 µM for 24 h, 48 h, and 72 h. Cell viability was estimated using the MTS assay. Data are represented as the mean ± SD as determined from triplicate experiments. A Student’s t-test was used to evaluate the data with the following significance levels: *p < 0.05, **p < 0.01, ***p < 0.001.

Next we determined the ability of the investigated compounds to affect cell proliferation of the tested cell lines. JB6 Cl41, HT-29, and MDA-MB-231 cells were treated with compounds **1**, **5**, and **6** at a non-toxic concentration of 20 µM for 24, 48, and 72 h. All tested compounds inhibited cell growth to a comparable degree (Figure 4). Compounds **1**, **5**, and **6** decreased proliferation of JB6 Cl41 cells by 20%, 22%, and 24%, respectively; HT-29 cells by 20%, 22%, and 26%, respectively; and MDA-MB-231 cells by 24%, 27%, and 29%, respectively, after 72 h of treatment.

#### 2.2.2. The Effect of Compounds **1**, **5**, and **6** on Colony Formation of Cancer Cells

**Figure 5:**
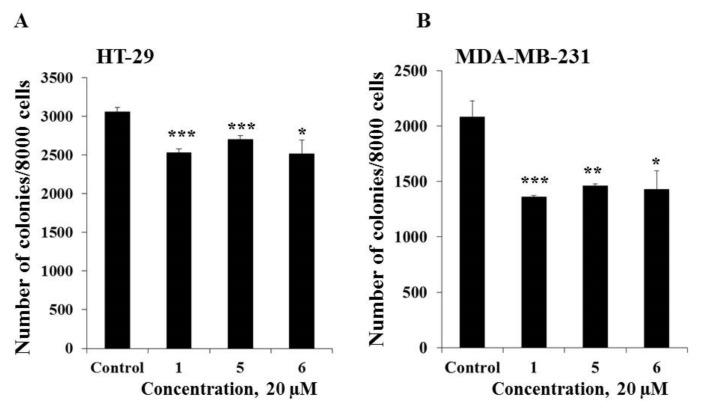
The effect of compounds **1**, **5**, and **6** on colony formation in human cancer cells. (**A**) HT-29; or (**B**) MDA-MB-231 cells (2.4 × 10^4^) with or without investigated compounds (20 µM) treatment were subcultured onto 0.3% Basal Medium Eagle (BME) agar containing 10% FBS, 2 mM L-glutamine, and 25 µg/mL gentamicin. After 14 days of incubation, the number of colonies was evaluated under a microscope with the aid of the ImageJ software program. Results are expressed as the mean ± standard deviation (SD). The asterisk (*) indicates a significant decrease in colony number of cancer cells treated by compounds as compared with the control (*p < 0.05, **p < 0.01, ***p < 0.001).

The effect of compounds **1**, **5**, and **6** on the colony formation of human cancer HT-29 and MDA-MB-231 cells was investigated using the soft agar assay. Compounds **1**, **5**, and **6** (20 µM) were demonstrated to possess comparable inhibiting activity on colony formation of cancer cells and the decrease in the colony number of HT-29 cells was by 18%, 12%, and 18%, respectively, while MDA-MB-231 cells was by 35%, 30%, and 31%, respectively as compared with non-treated cells (control) (Figure 5). In the present study it was demonstrated that triple negative human breast cancer cells MDA-MB-231 were more sensitive to the treatment of compounds **1**, **5**, and **6** than colorectal carcinoma cells HT-29.

In summary, compounds **1**, **5**, and **6** are non-cytototoxic against normal JB6 Cl41 and cancer HT-29 and MDA-MB-231 cell lines at concentrations up to 100 µM, however, they are able to suppress cell proliferation and colony formation of cancer HT-29 and MDA-MB-231 cells.

## 3. Experimental Section

### 3.1. General Procedures

The ^1^H- and ^13^C- NMR spectra were recorded on a Bruker Avance III 500 HD (Bruker, Germany) spectrometer at 500.13 and 125.76 MHz and a Bruker Avance III 700 spectrometer (Bruker, Germany) at 700.13 and 176.04 MHz, respectively. Chemical shifts (ppm) were internally referenced to the corresponding residual solvent signals δ_H_ 3.30/δ_C_ 49.0 for CD_3_OD. HRESIMS mass spectra were recorded on a Bruker Impact II Q-TOF mass spectrometer (Bruker, Bremen, Germany); the samples were dissolved in MeOH (c 0.001 mg/mL). Optical rotation was measured using the Perkin Elmer 343 polarimeter (Waltham, MA, USA). IR spectra were recorded on a Bruker OPUS Vector-22 infrared spectrophotometer. HPLC separations were carried out on an Agilent 1100 Series chromatograph (Agilent Technologies, Santa Clara,.CA, USA) equipped with a differential refractometer; Discovery C18 (5 µm, 250 × 10 mm, Supelco, Bellefonte, PA, USA) and YMC-Pack Pro C18 (5 µm, 250 × 4.6 mm, YMC CO., LTD, Kyoto, Japan) columns were used. Low pressure column liquid chromatography was performed using Amberlite XAD-2 (20 to 60 mesh, Supelco, Bellefonte, PA., USA), and silica gel KSK (50 to 160 µm, Sorbpolimer, Krasnodar, Russia). Sorbfil silica gel plates (4.5 × 6.0 cm, 5 to 17 μm, Sorbpolimer, Krasnodar, Russia) were used for thin-layer chromatography.

### 3.2. Animal Material

Specimens of *Anthenoides laevigatus* Liao & A.M. Clark, 1989 (order Valvatida, family Goniasteridae) were collected in January 2018 from the coastal waters of the Qui Nhon Province (Vietnam), at a depth of 20 to 30 m, and were identified by Dr. Do Cong Thung, the Institute of Marine Resources and Environment, VAST, Hai Phong, Vietnam. A voucher specimen (no. SBAL 01-2018 (was deposited at the Institute of Natural Products Chemistry, VAST, Vietnam.

### 3.3. Extraction and Isolation

The fresh animals (2.2 kg) were chopped into small pieces and extracted four times by MeOH with heating. The MeOH extract was evaporated *in vacuo*, and the residue (37 g) was dissolved in H_2_O (1.3 L). The H_2_O-soluble fraction was passed in two portions through an Amberlite XAD-2 column (7.5 × 28 cm) and eluted with distilled H_2_O until a negative chloride ion reaction was obtained, followed by elution with EtOH. The combined EtOH eluate was evaporated to give a brownish material (4.4 g). The resulting total fraction was chromatographed on a Si gel column (6.5× 15 cm) using CH_3_Cl-EtOH (stepwise gradient, 3:1→1:2, *v*/*v*), EtOH, and EtOH-H_2_O (stepwise gradient, 20:1→9:1, *v*/*v*) to give ten main fractions (1−10). Fractions 4 and 5 mainly contained the mixtures of polyhydroxylated steroids based on TLC data on Si gel plates in the eluent system toluene-EtOH (9:5, *v*/*v*). HPLC separation of fraction 4 (110 mg) on a Discovery C18 column with 55% aq. EtOH (1.5 mL/min) as an eluent system yielded pure **5** (4.1 mg, t_R_ 48.8 min) and subfraction 4.1 and 4.2 that were further purified on a YMC-Pack Pro C18 column with 78% aq. MeOH (0.9 mL/min) as an eluent system to give pure **2** (0.9 mg, t_R_ 15.3 min), **3** (0.9 mg, t_R_ 14.1 min), and **4** (1.1 mg, t_R_ 10.4 min). HPLC separation of fraction 5 (182 mg) on a Discovery C18 column with 55% aq. EtOH (1.5 mL/min) as an eluent system gave pure **1** (49.6 mg, t_R_ 21.0 min) and **6** (23.6 mg, t_R_ 26.1 min).

### 3.4. Compound Characterization Data

(25S)-5α-cholestane-3β,5,6β,14,15α,16β,26-heptaol (**1**): Colorless amorphous powder; [α${]}_{D}^{25}$ + 9.5 (*c* 0.8, MeOH); IR (KBr) ν_max_ 3401, 2943, 2870, 1385, 1048, 1017, 964 cm^−1^; (+)HRESIMS *m*/*z* 507.3292 [M + Na]^+^ (calcd for C_27_H_48_O_7_Na, 507.3292); (−)HRESIMS *m*/*z* 483.3321 [M – H]^–^ (calcd for C_27_H_47_O_7_, 483.3327); ^1^H-NMR data, see Table 1; ^13^C-NMR data, see Table 2.

(25S)-5α-cholestane-3β,6β,14,15α,16β,26-hexaol (**2**): Colorless amorphous powder; [α${]}_{D}^{25}$ + 14.4 (*c* 0.1, MeOH); (+)HRESIMS *m*/*z* 491.3337 [M + Na]^+^ (calcd for C_27_H_48_O_6_Na, 491.3343); (−)HRESIMS *m*/*z* 467.3379 [M – H]^–^ (calcd for C_27_H_47_O_6_, 467.3378); ^1^H-NMR data, see Table 1; ^13^C-NMR data, see Table 2.

(25S)-5β-cholestane-3β,6β,14,15α,16β,26-hexaol (**3**): Colorless amorphous powder; [α${]}_{D}^{25}$ + 13.3 (*c* 0.1, MeOH); (+)HRESIMS *m/z* 491.3341 [M + Na]^+^ (calcd for C_27_H_48_O_6_Na 491.3343); (−)HRESIMS *m*/*z* 467.3380 [M – H]^–^ (calcd for C_27_H_47_O_6_, 467.3378); ^1^H-NMR data, see Table 1; ^13^C-NMR data, see Table 2.

(25S)-5β-cholestane-3β,4α,6β,14,15α,16β,26-heptaol (**4**): Colorless amorphous powder; [α${]}_{D}^{25}$ + 15.5 (*c* 0.1, MeOH); (+)HRESIMS *m*/*z* 507.3290 [M + Na]^+^ (calcd for C_27_H_48_O_7_Na, 507.3292); (−)HRESIMS *m*/*z* 483.3327 [M – H]^–^ (calcd for C_27_H_47_O_7_, 483.3327); ^1^H-NMR data, see Table 1; ^13^C-NMR data, see Table 2.

### 3.5. Preparation of the MTPA Esters of Compound **1**

Aliquots (0.7 mg each) of compound **1** were treated with *S*-(+)- and *R*-(–)-α-methoxy-α-(trifluoromethyl)-phenylacetyl (MTPA) chloride (0.7 μL) in dry pyridine (180 μL) for 3 h at room temperature. After removal of the solvent, the products were purified on a Si gel column (0.8 × 5 cm) using CHCl_3_-EtOH (50:1, *v*/*v*) to obtain the corresponding (*R*)- and (*S*)-MTPA esters of **1**.

3,15,26-tri-(*R*)-MTPA ester of **1**: Selected ^1^H-NMR (500.13 MHz, CD_3_OD): *δ*_H_ 0.75 (1H, m, H-7), 0.87 (3H, d, *J* = 6.7 Hz, H_3_-21), 0.91 (3H, d, *J* = 6.8 Hz, H_3_-27), 1.07 (3H, s, H_3_-18), 1.12 (3H, s, H_3_-19), 1.49 (1H, m, H′-7), 1.83 (1H, m, H-25), 2.10 (1H, m, H-17), 3.05 (1H, dd, *J* = 3.6, 2.5 Hz, H-6), 4.15 (1H, m, H-16), 4.15 (1H, dd, *J* = 10.7, 5.7 Hz, H-26), 4.17 (1H, dd, *J* = 10.7, 6.0 Hz, H′-26), 5.03 (1H, d, *J* = 3.1 Hz, H-15), 5.39 (1H, m, H-3).

3,6,15,26-tetra-(*S*)-MTPA ester of **1**: Selected ^1^H-NMR (500.13 MHz, CD_3_OD): *δ*_H_ 0.62 (3H, s, H_3_-19), 0.87 (3H, d, *J* = 6.7 Hz, H_3_-21), 0.90 (3H, d, *J* = 6.8 Hz, H_3_-27), 1.02 (3H, s, H_3_-18), 1.33 (1H, m, H-7), 1.82 (1H, m, H-25), 2.02 (1H, dd, *J* = 11.2, 8.2 Hz, H-17), 2.09 (1H, m, H′-7), 4.08 (2H, m, H-16, H-26), 4.09 (1H, m, H-16), 4.24 (1H, dd, *J* = 10.7, 5.5 Hz, H′-26), 4.83 (1H, t, *J* = 3.0 Hz, H-6), 5.19 (1H, d, *J* = 3.4 Hz, H-15), 5.32 (1H, m, H-3).

### 3.6. Bioactivity Assay

#### 3.6.1. Reagents

Phosphate buffered saline (PBS), L-glutamine, penicillin-streptomycin solution (10,000 U/mL, 10 µg/mL) were from Sigma-Aldrich company (St. Louis, MO, USA). MTS reagent (3-(4,5-dimethylthiazol-2-yl)-5-(3-carboxymethoxyphenyl)-2-(4-sulfophenyl)-2H-tetrazolium) was purchased from Promega (Madison, Wisconsin, USA). The Basal Medium Eagle (BME), Dulbecco’s modified Eagle’s medium (DMEM), and McCoy’s 5A modified medium (McCoy’s 5A), trypsin, fetal bovine serum (FBS), and agar were purchased from ThermoFisher Scientific (Waltham, MA, USA).

#### 3.6.2. Cell Lines and Culture Conditions

Mouse epidermal cells JB6 Cl41 (ATCC^®^ no. CRL-2010™), human colorectal carcinoma HT-29 (ATCC^®^ no. HTB-38^™^), and human breast adenocarcinoma MDA-MB-231 (ATCC^®^ HTB-26^™^) cells were obtained from the American Type Culture Collection (Manassas, VA, USA).

JB6 Cl41 cells grown in MEM supplemented with 5% fetal bovine serum (FBS), HT-29 cells were cultured in McCoy’s 5A with 10% FBS, and MDA-MB-231 cells were grown in DMEM with 10% FBS. The cell cultures were maintained at 37 °C in humidified atmosphere containing 5% CO_2_.

#### 3.6.3. MTS Assay

To determine the cytotoxic activity of compounds **1**, **5**, and **6**, JB6 Cl41, HT-29, and MDA-MB-231 cells were seeded at a density of 1.0 × 10^4^cells/200 µL of complete MEM/5% FBS, McCoy’s 5A/10% FBS, and DMEM/10% FBS media, respectively, in 96-well plates. After incubation for 24 h, the cells were treated with tested compounds in the range of concentration 5–100 µM, while the control was treated with the complete medium only. Cells were cultured for additional 24 h at 37 °C in 5% CO_2_ atmosphere. Subsequently, MTS reagent (20 µL) was added to each well, and the cells were incubated for an additional 3 h at 37 °C in 5% CO_2_. Absorbance was measured at 490/630 nm by a Power Wave XS microplate reader (BioTek, Winooski, VT, USA). All tested samples were carried out in triplicates. Compound’s concentration causing 50% of cell viability inhibition (IC_50_) were calculated.

To analyze the anti-proliferative activity of compounds **1**, **5**, and **6**, the cells (1.0 × 10^4^cells/200 µL) were treated with tested compounds at concentration of 20 µM and incubated for an additional 24, 48, and 72 h at 37 °C in 5% CO_2_. MTS reagent (20 µL) was added to each well, and the cells were incubated for an additional 3 h at 37 °C in 5% CO_2_. Absorbance was measured at 490/630 nm using a microplate reader. All tested samples were analyzed in triplicates.

#### 3.6.4. Soft Agar Assay

Cells (2.4 × 10^4^/mL) were grown in 1 mL of 0.3% Basal Medium Eagle’s agar containing 10% FBS. The cells were treated by compounds **1**, **5**, and **6** at non-toxic concentration of 5, 10, and 20 µM. The cultures were maintained at 37 °C in 5% CO_2_ incubator for 2 weeks and the number and size of the colonies were determined using a Motic microscope AE 20 (XiangAn, Xiamen, China) and ImageJ software bundled with 64-bit Java 1.8.0_112 (NIH, Bethesda, Maryland, USA).

#### 3.6.5. Statistical Analysis

Results are expressed as the mean ± standard deviation (SD). Student’s T test was used to evaluate the data with the following significance levels: *p < 0.05, **p < 0.01, ***p < 0.001. All assays were performed in at least three independent experiments.

## 4. Conclusions

Four new polyhydroxylated steroids were isolated along with two previously known related steroids from the Vietnamese starfish *A. laevigatus* and their chemical structures were elucidated. Two new compounds have the common 5*α*-cholestane skeleton, while the other two new compounds have the 5*β*-cholestane skeleton, which are rare among starfish steroids. Previously, only two steroid compounds with the *cis*-A/B ring junction were known from two species of the starfish *L. clathrata* and *T. novaecaledoniae*. Three of the substances that were isolated from *A. laevigatus* did not show cytotoxic effects against normal JB6 Cl41 and human colorectal cancer HT-29 and breast cancer MDA-MB-231 cells, however they suppressed cell proliferation and colony formation of cancer HT-29 and MDA-MB-231 cells.

## Figures and Tables

**Table 1:** ^1^H-NMR data of compounds **1**−**4** (CD_3_OD, *δ* in ppm, *J* in Hz) *^a^*.

Position	1	2	3	4
1	1.62, m 1.32, m	1.63, m 0.98, m	1.45, m 1.39, m	1.47, m
2	1.75, m 1.47, m	1.73, m 1.40, m	1.56, m 1.44, m	1.88, m 1.32, m
3	4.00, m	3.53, m	3.99, br. q (2.7)	3.70, br. q (3.7)
4	2.04, dd (13.0, 11.3) 1.53, m	1.72, m 1.53, m	1.66, m 1.38, m	3.66, t (3.7)
5	‒	1.09, dt (13.0, 2.6)	1.83, m	1.69, t (3.3)
6	3.49, t (3.0)	3.77, q (2.6)	3.66, q (2.7)	4.00, q (3.3)
7	1.69, ddd (14.2, 3.9, 2.6) 2.03, m	1.94, m 1.50, m	1.74, m 1.66, m	2.19, m 1.74, m
8	2.22, td (12.4, 4.0)	2.22, td (12.4, 3.5)	2.25, td (11.7, 4.0)	2.21, m
9	1.91, m	1.28, m	1.98, m	2.11, m
10	‒	‒	‒	‒
11	1.33, m	1.44, m	1.35, m	1.37, m
12	1.81, m	1.78, m 1.60, m	1.80, m 1.61, m	1.77, m 1.59, m
13	‒	‒	‒	‒
14	‒	‒	‒	‒
15	3.86, d (3.3)	3.89, d (3.3)	3.89, d (3.2)	3.87, d (3.2)
16	3.96, dd (8.1, 3.3)	3.96, dd (7.9, 3.3)	3.97, dd (8.0, 3.2)	3.96, dd (8.0, 3.2)
17	1.96, dd (11.3, 8.1)	1.96, t (10.9)	1.96, m	1.95, dd (11.0, 8.0)
18	1.00, s	1.01, s	1.00, s	1.00, s
19	1.17, s	1.04, s	1.13, s	1.10, s
20	1.80, m	1.80, m	1.80, m	1.79, m
21	0.90, d (6.7)	0.90, d (6.7)	0.90, d (6.8)	0.90, d (6.5)
22	1.58, m 1.02, m	1.58, m 1.02, m	1.58, m 1.02, m	1.58, m 1.01, m
23	1.47, m 1.22, m	1.47, m 1.21, m	1.48, m 1.22, m	1.47, m 1.21, m
24	1.41, m 1.04, m	1.41, m 1.04, m	1.42, m 1.05, m	1.41, m 1.05, m
25	1.56, m	1.57, m	1.56, m	1.56 m
26	3.42, dd (10.5, 5.7) 3.30, m	3.42, dd (10.9, 6.0) 3.30, m	3.42, dd (10.7, 5.8) 3.30, m	3.41, dd (10.5, 6.0) 3.30, m
27	0.90, d (6.6)	0.90, d (6.7)	0.90, d (6.8)	0.90, d (6.7)

*^a^* Assignments from 700 MHz COSY, HSQC, HMBC (8 Hz), and ROESY (250 ms) data.

**Table 2:** ^13^C-NMR data of compounds **1**−**4** (CD_3_OD).

Position	1	2	3	4
1	33.7	40.0	31.5	31.3
2	31.7	32.2	28.1	24.4
3	68.3	72.4	67.1	72.2
4	41.4	36.4	34.4	75.3
5	76.6	48.7	44.0	48.4
6	76.5	72.8	74.3	73.3
7	30.3	35.6	29.9	33.3
8	34.6	34.7	35.0	34.8
9	39.1	47.8	33.4	37.1
10	39.5	36.7	36.0	36.5
11	21.1	20.9	20.7	21.3
12	33.7	33.5	33.6	33.6
13	48.2	48.2	48.2	48.1
14	82.7	82.7	82.8	82.8
15	85.6	85.6	85.5	85.8
16	82.1	82.1	82.2	82.1
17	54.4	54.5	54.5	54.5
18	16.5	16.4	16.4	16.4
19	17.3	16.1	26.3	25.6
20	31.0	31.0	31.0	31.0
21	18.7	18.7	18.7	18.7
22	37.9	37.9	37.9	37.9
23	25.1	25.1	25.1	25.1
24	34.9	34.9	34.9	34.9
25	37.0	37.0	37.0	37.0
26	68.4	68.4	68.4	68.4
27	17.3	17.3	17.3	17.3

## Supplementary Materials

The following are available online. Copies HRESIMS (Figures S1, S9, S16, and S23), ^1^H-NMR (Figures S2, S10, S17, and S24), ^13^C-NMR (Figures S3, S11, S18, and S25), COSY (Figures S4, S12, S19, and S26), HSQC (Figures S5, S13, 20, and S27), HMBC (Figures S6, S14, S21, and S28), and ROESY (Figures S7, S15, S22, and S29) spectra of compounds **1**, **2**, **3**, and **4**, respectively. Copies ^1^H-NMR (Figure S8) spectra of (*R*)- and (*S*)-MTPA esters of **1**, 1D TOCSY (Figure S30) spectrum of **4**.

## References

[1] Minale L., Riccio R., Zollo F. (1993). Steroidal oligoglycosides and polyhydroxysteroids from Echinoderms. Chem. Org. Nat..

[2] Stonik V.A. (2001). Marine polar steroids. Russ. Chem. Rev..

[3] Iorizzi M., De Marino S., Zollo F. (2001). Steroidal oligoglycosides from the Asteroidea. Curr. Org. Chem..

[4] Stonik V.A., Ivanchina N.V., Kicha A.A. (2008). New polar steroids from starfish. Nat. Prod. Commun..

[5] Dong G., Xu T.H., Yang B., Lin X.P., Zhou X.F., Yang X.W., Liu Y.H. (2011). Chemical constituents and bioactivities of starfish. Chem. Biodivers..

[6] Ivanchina N.V., Kicha A.A., Stonik V.A. (2011). Steroid glycosides from marine organisms. Steroids.

[7] Ivanchina N.V., Kicha A.A., Malyarenko T.V., Stonik V.A., Gomes A.R., Rocha-Santos T., Duarte A. (2017). Advances in Natural Products Discovery.

[8] Gomes A.R., Freitas A.C., Rocha-Santos T.A.P., Duarte A.C. (2014). Bioactive compounds derived from echinoderms. Rsc Adv..

[9] Lazzara V., Arizza V., Luparello C., Mauro M., Vazzana M. (2019). Bright spots in the darkness of cancer: A review of starfishes-derived compounds and their anti-tumor action. Mar. Drugs.

[10] Malyarenko O.S., Malyarenko T.V., Kicha A.A., Ivanchina N.V., Ermakova S.P. (2019). Effects of polar steroids from the starfish *Patiria (=Asterina) pectinifera* in combination with X-ray radiation on colony formation and apoptosis induction of human colorectal carcinoma cells. Molecules.

[11] Ha D.T., Kicha A.A., Kalinovsky A.I., Malyarenko T.V., Popov R.S., Malyarenko O.S., Ermakova S.P., Thuy T.T.T., Long P.Q., Ivanchina N.V. (2019). Asterosaponins from the tropical starfish Acanthaster planci and their cytotoxic and anticancer activities in vitro. Nat. Prod. Res..

[12] Kicha A.A., Ha D.T., Ivanchina N.V., Malyarenko T.V., Kalinovsky A.I., Dmitrenok P.S., Ermakova S.P., Malyarenko O.S., Hung N.A., Thuy T.T.T. (2018). Six new polyhydroxysteroidal glycosides, anthenosides S1−S6, from the starfish *Anthenea Sibogae*. Chem. Biodivers..

[13] Malyarenko T.V., Ivanchina N.V., Malyarenko O.S., Kalinovsky A.I., Dmitrenok P.S., Evtushenko E.V., Minh C.V., Kicha A.A. (2018). Two new steroidal monoglycosides, anthenosides A_1_ and A_2_, and revision of the structure of known anthenoside A with unusual monosaccharide residue from the starfish. Anthenea Aspera Mol..

[14] Malyarenko T.V., Kharchenko S.D., Kicha A.A., Ivanchina N.V., Dmitrenok P.S., Chingizova E.A., Pislyagin E.A., Evtushenko E.V., Antokhina T.I., Minh C.V. (2016). Anthenosides L‒U, steroidal glycosides with unusual structural features from the starfish *Anthenea aspera*. J. Nat. Prod..

[15] Kicha A.A., Kalinovsky A.I., Malyarenko T.V., Ivanchina N.V., Dmitrenok P.S., Menchinskaya E.S., Yurchenko E.A., Pislyagin E.A., Aminin D.L., Huong T.T.T. (2015). Cyclic steroid glycosides from the starfish *Echinaster luzonicus*: Structures and immunomodulatory activities. J. Nat. Prod.

[16] Iorizzi M., Bryan P., McClintock J., Minale L., Palagiano E., Maurelli S., Riccio R., Zollo F. (1995). Chemical and biological investigation of the polar constituents of the starfish *Luidia clathrata*, collected in the gulf of Mexico. J. Nat. Prod.

[17] Ivanchina N.V., Kicha A.A., Kalinovsky A.I., Dmitrenok P.S., Dmitrenok A.S., Chaikina E.L., Stonik V.A., Gavagnin M., Cimino G. (2006). Polar steroidal compounds from the Far Eastern starfish *Henricia leviuscula*. J. Nat. Prod..

[18] Minale L., Pizza C., Riccio R., Squillace-Greco O., Zollo F., Pusset J., Menou J.L. (1984). New polyhydroxylated sterols from the starfish Luidia Maculate. J. Nat. Prod..

[19] Vanderach D.J., Djerassi C. (1978). Marine natural products. Synthesis of four naturally occurring 20β-H cholanic acid derivatives. J. Org. Chem..

[20] De Riccardis F., Minale L., Riccio R., Giovannitti B., Iorizzi M., Debitus C. (1993). Phosphated and sulfated marine polyhydroxylated steroids from the starfish *Tremaster Novaecaledoniae*. Gazz. Chim. Ital..

[21] Kicha A.A., Ivanchina N.V., Malyarenko T.V., Kalinovsky A.I., Popov R.S., Stonik V.A. (2019). Six new polyhydroxylated steroids conjugated with taurine, microdiscusols A-F, from the Arctic starfish *Asterias Microdiscus*. Steroids.

