# Supplementary material for: Unusual Polyhydroxylated Steroids from the Starfish Anthenoides laevigatus, Collected off the Coastal Waters of Vietnam
Source: Molecules. 2020 Mar 23;25(6):1440. doi: 10.3390/molecules25061440 (PMC7145304; doi:10.3390/molecules25061440)

Supplementary Data

Unusual Polyhydroxylated Steroids from the Starfish *Anthenoides laevigatus*, Collected off the Coastal Waters of Vietnam

Alla A. Kicha [1,*], Dinh T. Ha [3,4], Timofey V. Malyarenko [1,2], Anatoly I. Kalinovsky [1], Roman S. Popov [1], Olesya S. Malyarenko [1], Tran T. T. Thuy [3,*], Pham Q. Long [3], Nguyen T. T. Ha [5] and Natalia V. Ivanchina [1]

[1] G.B. Elyakov Pacific Institute of Bioorganic Chemistry, Far Eastern Branch of Russian Academy of Sciences, Pr. 100-let Vladivostoku 159, 690022 Vladivostok, Russia; ivanchina@piboc.dvo.ru (N.V.I.); malyarenko-tv@mail.ru (T.V.M.); kaaniv@piboc.dvo.ru (A.I.K.); prs_90@mail.ru (R.S.P.); malyarenko.os@gmail.com (O.S.M.)

[2] School of Natural Sciences, Far Eastern Federal University, Sukhanova Str. 8, Vladivostok 690000, Russia

[3] Institute of Natural Products Chemistry, Vietnam Academy of Science and Technology, 18 Hoang Quoc Viet, Cau Giay, Hanoi, Vietnam; dinhha.inpc@gmail.com (D.T.H.); mar.biochem@fpt.vn (P.Q.L.)

[4] Graduate University of Science and Technology, Vietnam Academy of Science and Technology, 18 Hoang Quoc Viet, Cau Giay, Hanoi, Vietnam

[5] Institute of Chemistry, Vietnam Academy of Science and Technology, 18 Hoang Quoc Viet, Cau Giay, Hanoi, Vietnam; thuha.vast@gmail.com (N.T.T.H)

* Correspondence: kicha@piboc.dvo.ru, allakicha@mail.ru; Tel.: +7-423-2312-360; Fax: +7-423-2314-050 (A.A.K.); thuytran.inpc@gmail.com (T.T.T.T.)

List

	Page
**Figure S1.** HRESIMS Spectrum of Compound **1**	3
**Figure S2.** ^{1}H-NMR (700.13 MHz, CD_3OD) Spectrum of Compound **1**	4
**Figure S3.** ^{13}C-NMR (176.07 MHz, CD_3OD) Spectrum of Compound **1**	5
**Figure S4.** COSY (700.13 MHz, CD_3OD) Spectrum of Compound **1**	6
**Figure S5.** HSQC (700.13 MHz, CD_3OD) Spectrum of Compound **1**	7
**Figure S6.** HMBC (700.13 MHz, CD_3OD) Spectrum of Compound **1**	8
**Figure S7.** ROESY (700.13 MHz, CD_3OD) Spectrum of Compound **1**	9
**Figure S8.** ^{1}H-NMR (700.13 MHz, CD_3OD) Spectra of (*R*)- and (*S*)-MTPA esters of **1**	10
**Figure S9.** HRESIMS Spectrum of Compound **2**	11
**Figure S10.** ^{1}H-NMR (700.13 MHz, CD_3OD) Spectrum of Compound **2**	12
**Figure S11.** ^{13}C-NMR (176.07 MHz, CD_3OD) Spectrum of Compound **2**	13
**Figure S12.** COSY (700.13 MHz, CD_3OD) Spectrum of Compound **2**	14
**Figure S13.** HSQC (700.13 MHz, CD_3OD) Spectrum of Compound **2**	15
**Figure S14.** HMBC (700.13 MHz, CD_3OD) Spectrum of Compound **2**	16
**Figure S15.** ROESY (700.13 MHz, CD_3OD) Spectrum of Compound **2**	17
**Figure S16.** HRESIMS Spectrum of Compound **3**	18
**Figure S17.** ^{1}H-NMR (700.13 MHz, CD_3OD) Spectrum of Compound **3**	19
**Figure S18.** ^{13}C-NMR (176.07 MHz, CD_3OD) Spectrum of Compound **3**	20
**Figure S19.** COSY (700.13 MHz, CD_3OD) Spectrum of Compound **3**	21
**Figure S20.** HSQC (700.13 MHz, CD_3OD) Spectrum of Compound **3**	22
**Figure S21.** HMBC (700.13 MHz, CD_3OD) Spectrum of Compound **3**	23
**Figure S22.** ROESY (700.13 MHz, CD_3OD) Spectrum of Compound **3**	24
**Figure S23.** HRESIMS Spectrum of Compound **4**	25
**Figure S24.** ^{1}H-NMR (700.13 MHz, CD_3OD) Spectrum of Compound **4**	26
**Figure S25.** ^{13}C-NMR (176.07 MHz, CD_3OD) Spectrum of Compound **4**	27
**Figure S26.** COSY (700.13 MHz, CD_3OD) Spectrum of Compound **4**	28
**Figure S27.** HSQC (700.13 MHz, CD_3OD) Spectrum of Compound **4**	29
**Figure S28.** HMBC (700.13 MHz, CD_3OD) Spectrum of Compound **4**	30
**Figure S29.** ROESY (700.13 MHz, CD_3OD) Spectrum of Compound **4**	31
**Figure S30.** 1D TOCSY (700.13 MHz, CD_3OD) Spectrum of Compound **4**	32

Figure S1. HRESIMS Spectrum of Compound **1**

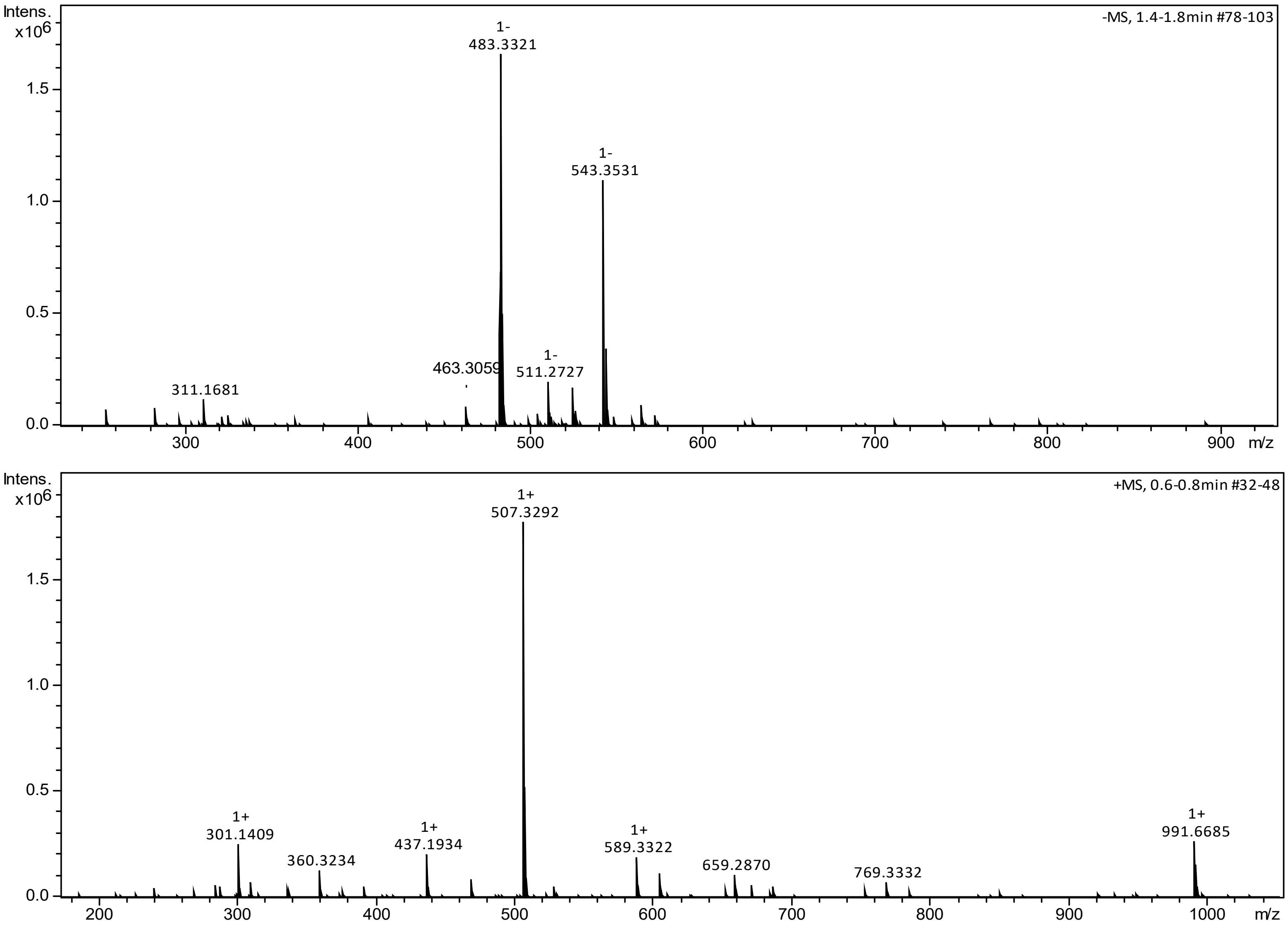

Figure S2. ^{1}H-NMR (700.13 MHz, CD_3OD) Spectrum of Compound **1**.

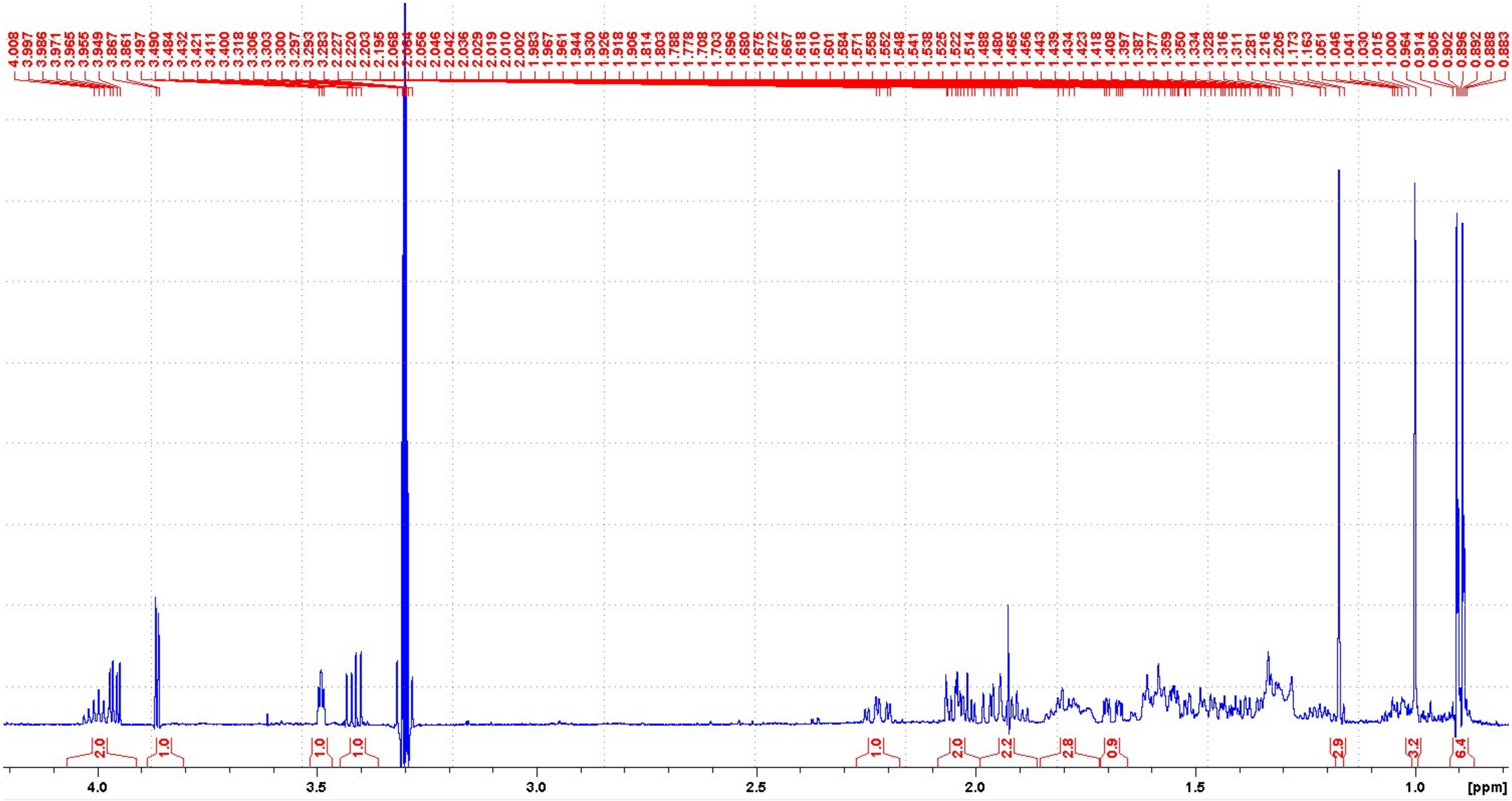

Figure S3. ^{13}C-NMR (176.07 MHz, CD_3OD) Spectrum of Compound **1**.

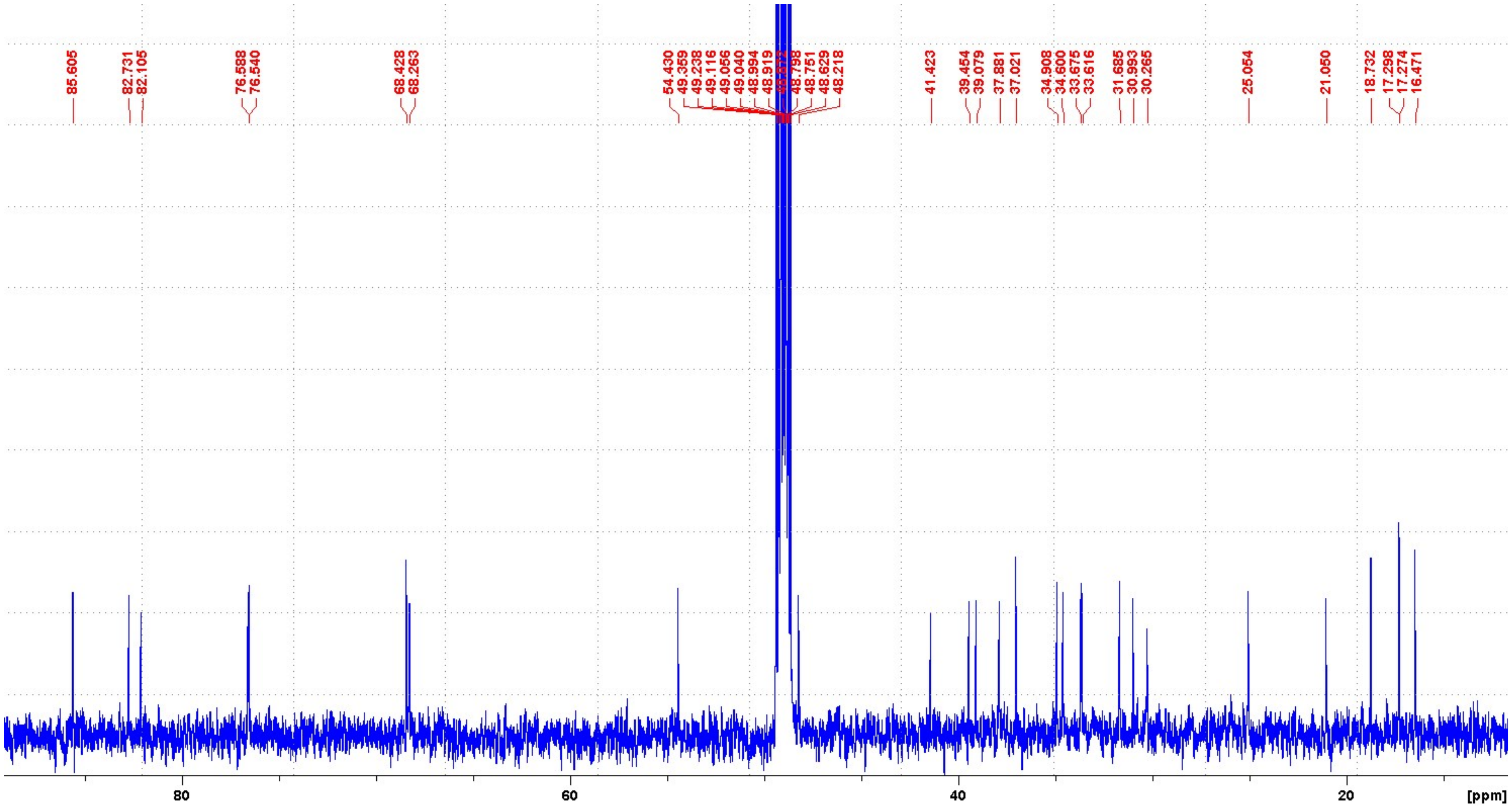

Figure S4. COSY (700.13 MHz, CD_3OD) Spectrum of Compound **1**.

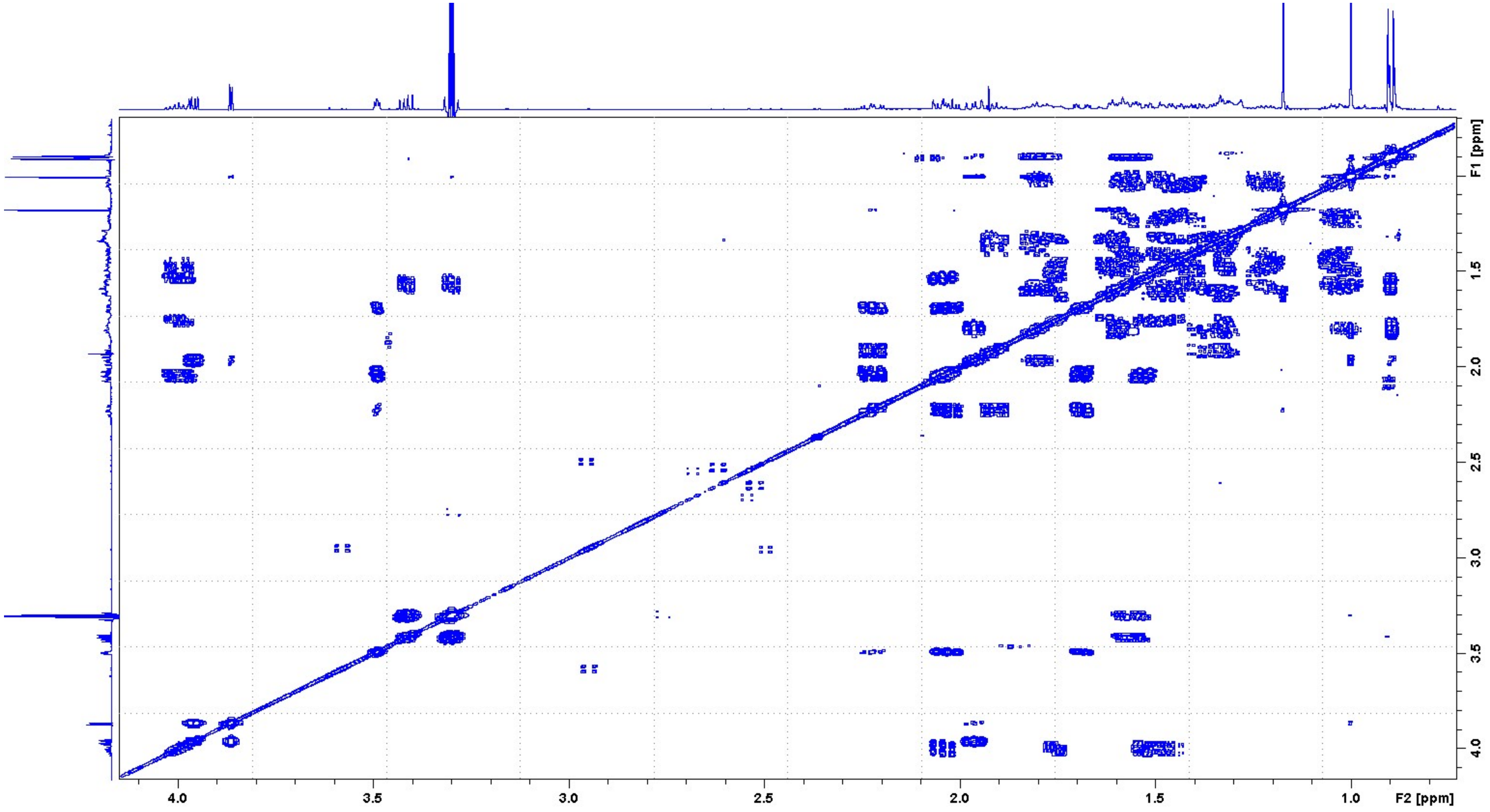

Figure S5. HSQC (700.13 MHz, CD_3OD) Spectrum of Compound **1**.

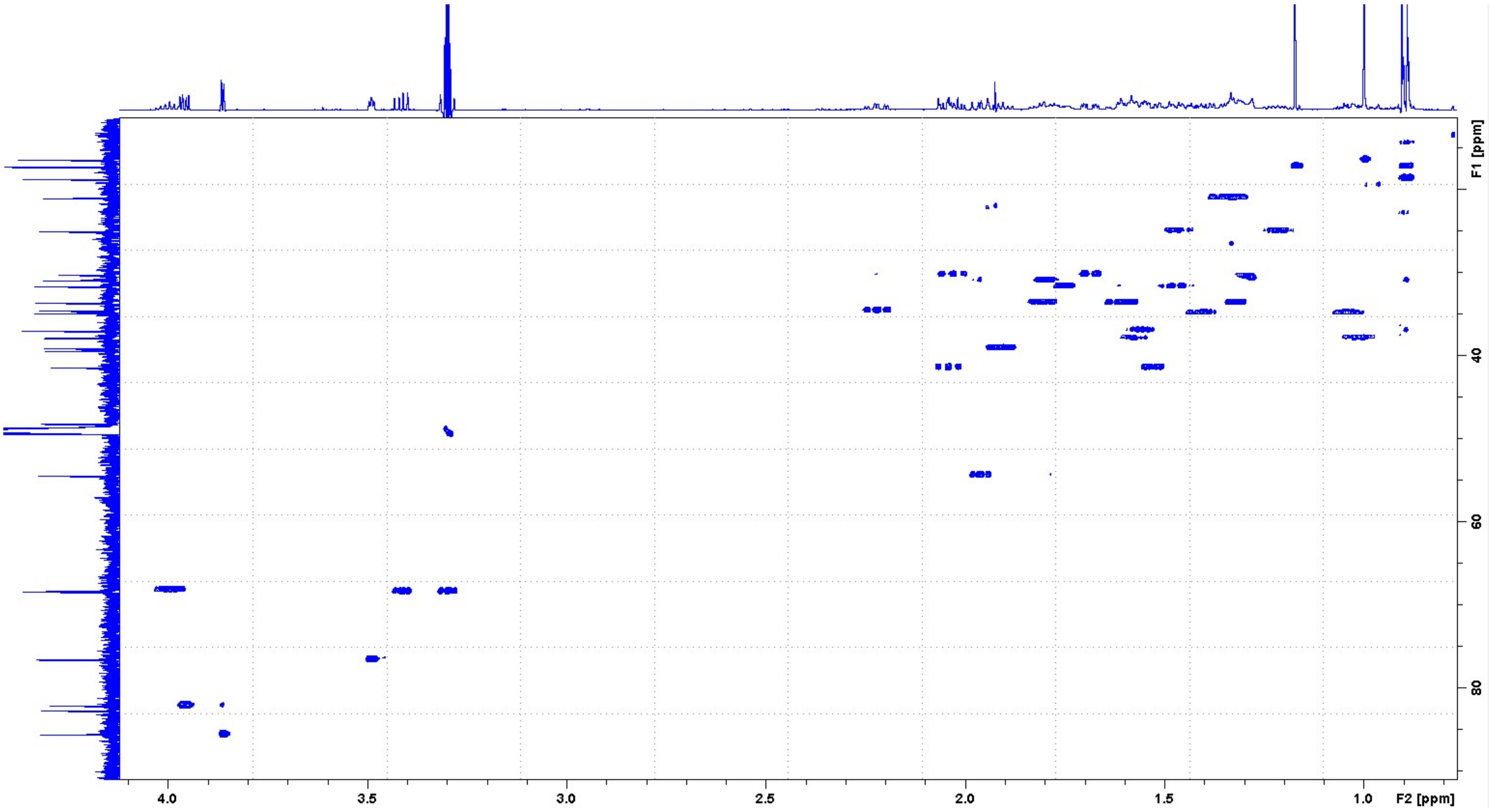

Figure S6. HMBC (700.13 MHz, CD_3OD) Spectrum of Compound **1**.

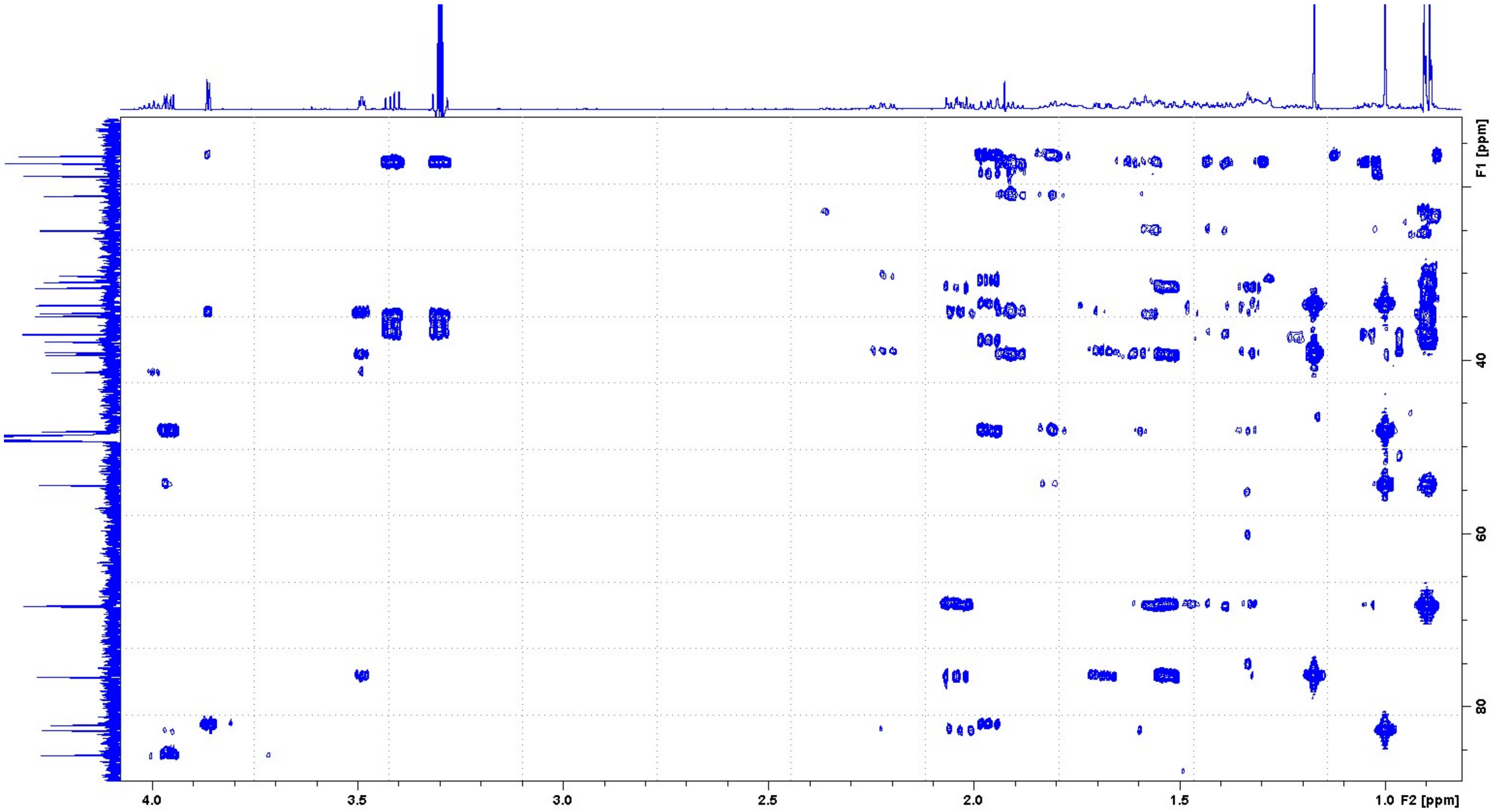

Figure S7. ROESY (700.13 MHz, CD_3OD) Spectrum of Compound **1**.

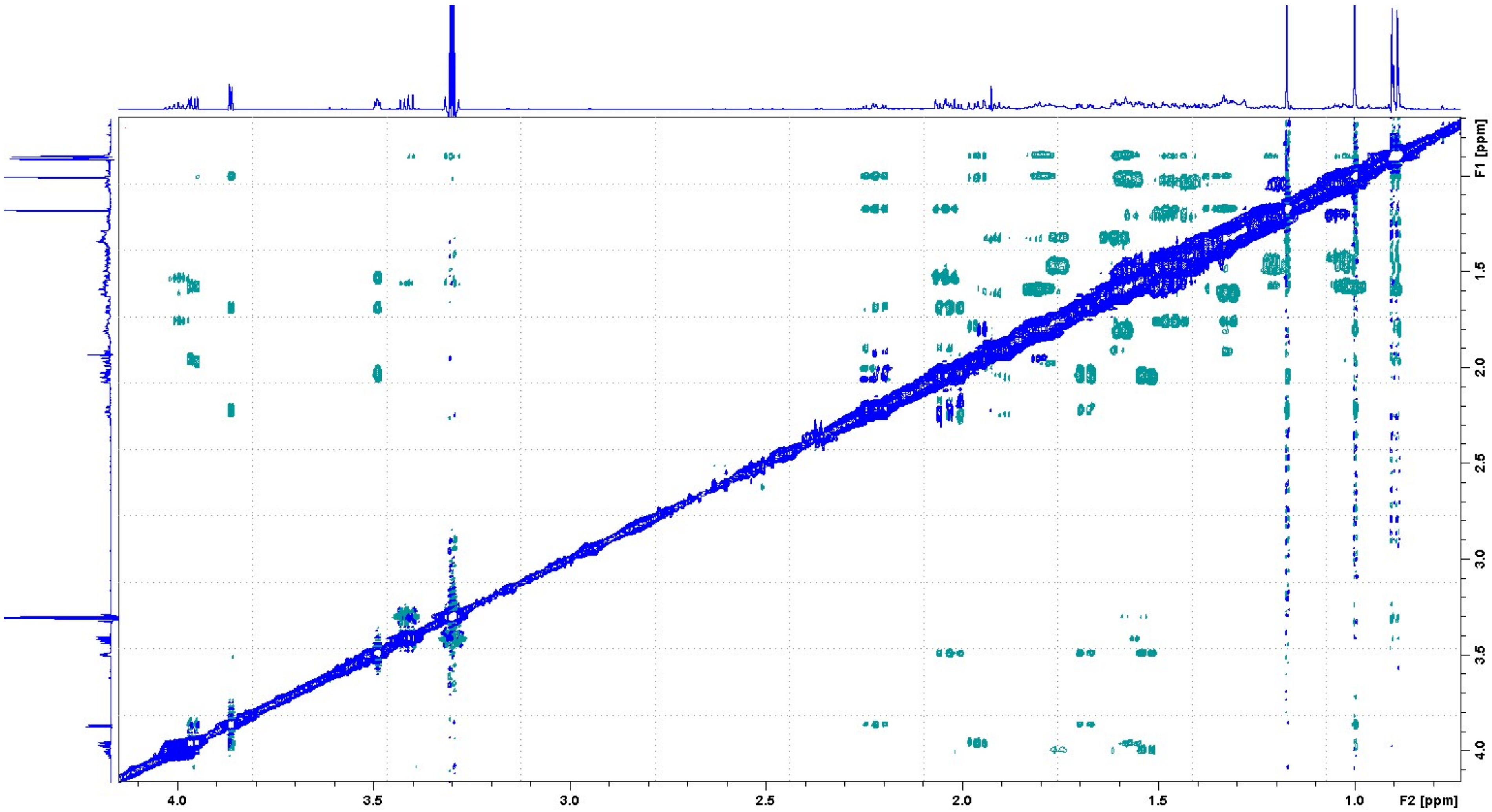

Figure S8. ^{1}H-NMR (700.13 MHz, CD_3OD) Spectra of (*R*)- and (*S*)-MTPA esters of **1**

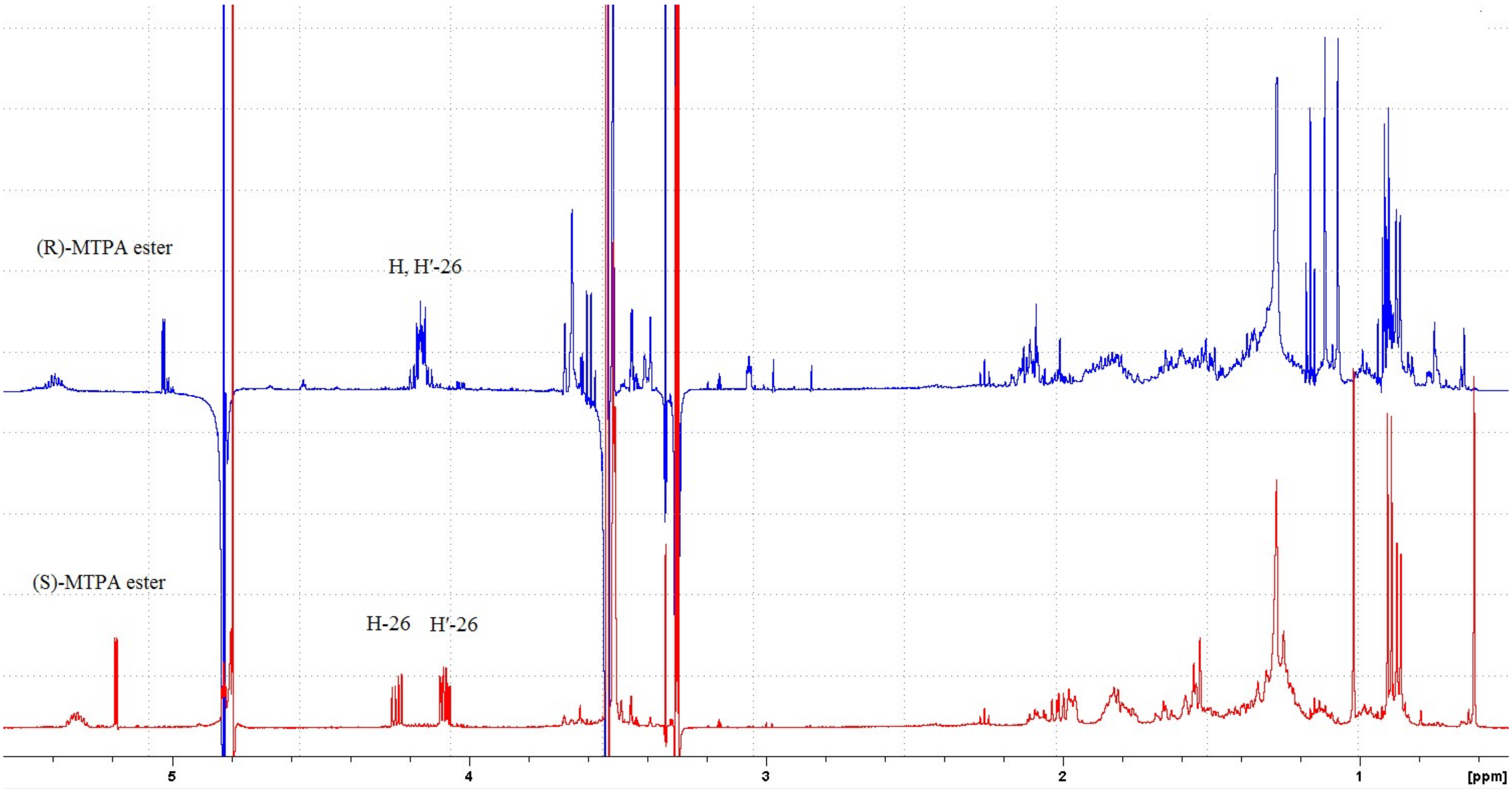

Figure S9. HRESIMS Spectrum of Compound **2**

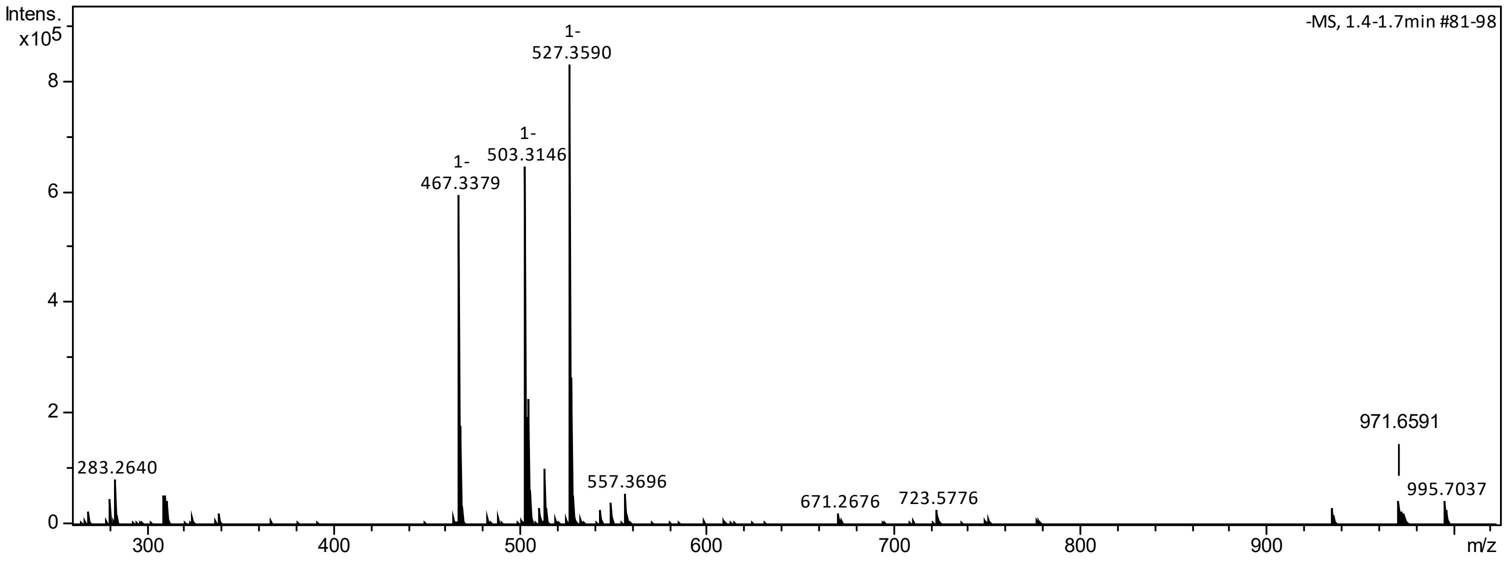

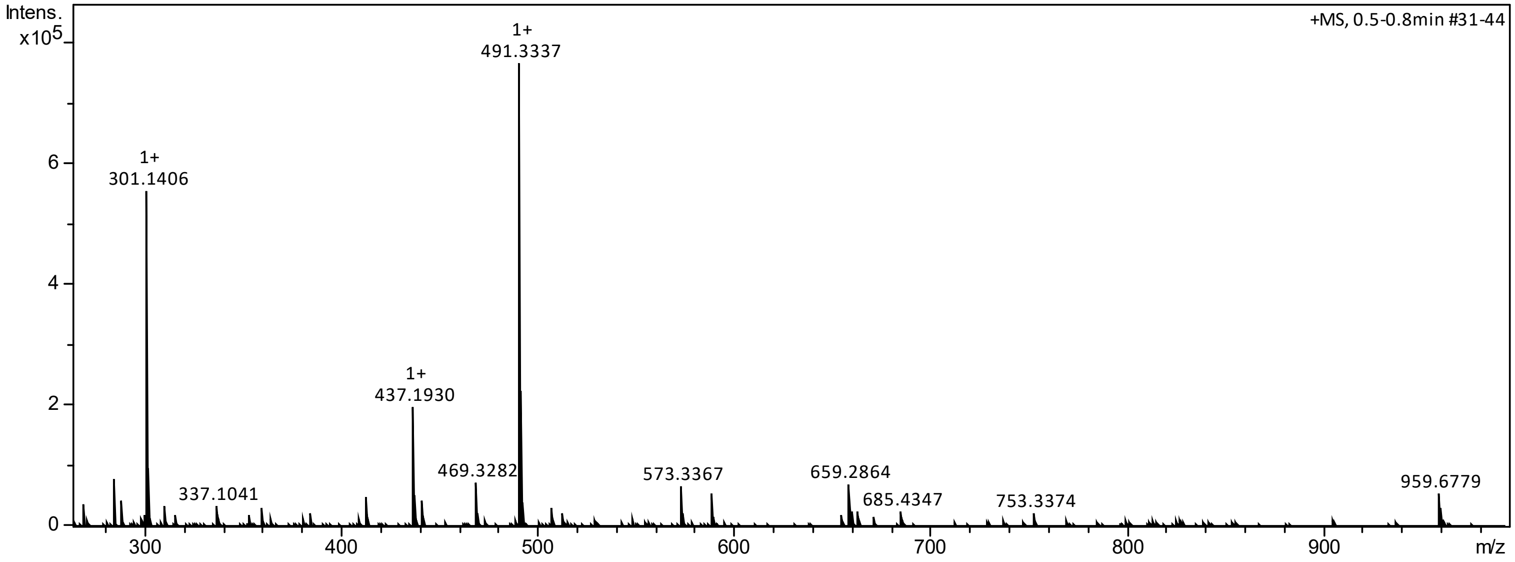

Figure S10. ^{1}H-NMR (700.13 MHz, CD_3OD) Spectrum of Compound **2**

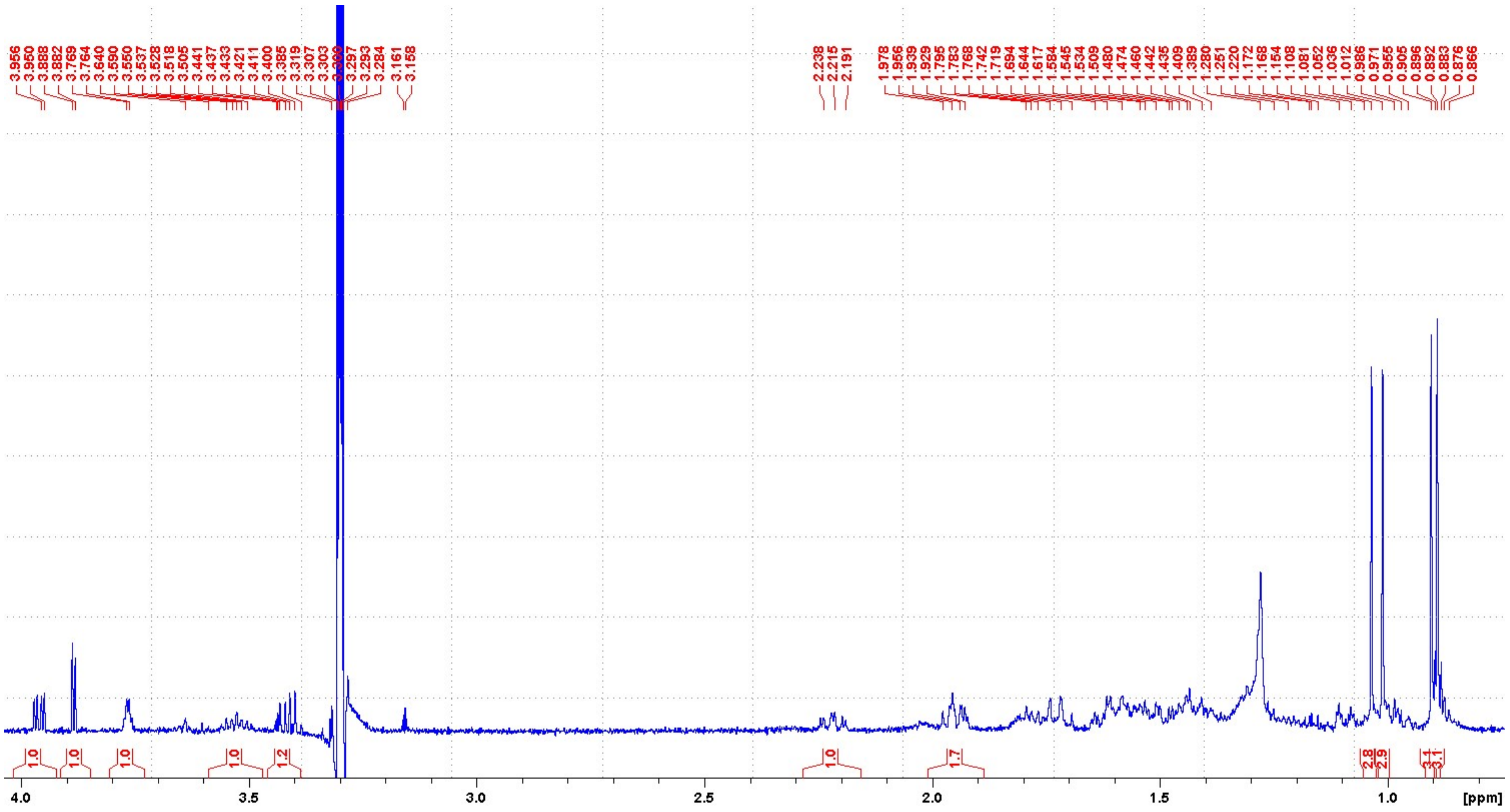

Figure S11. ^{13}C-NMR (176.07 MHz, CD_3OD) Spectrum of Compound **2**

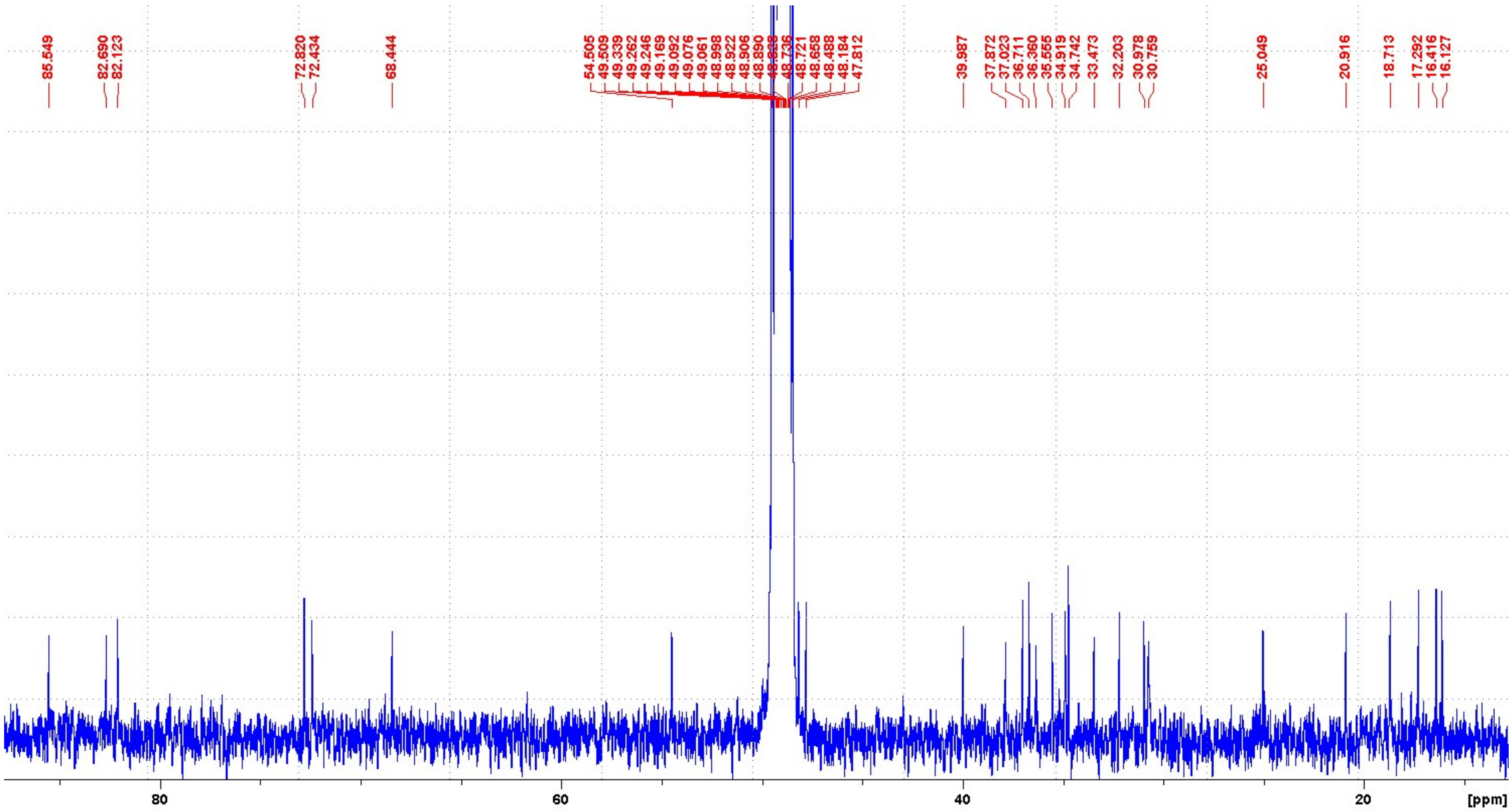

Figure S12. COSY (700.13 MHz, CD_3OD) Spectrum of Compound **2**

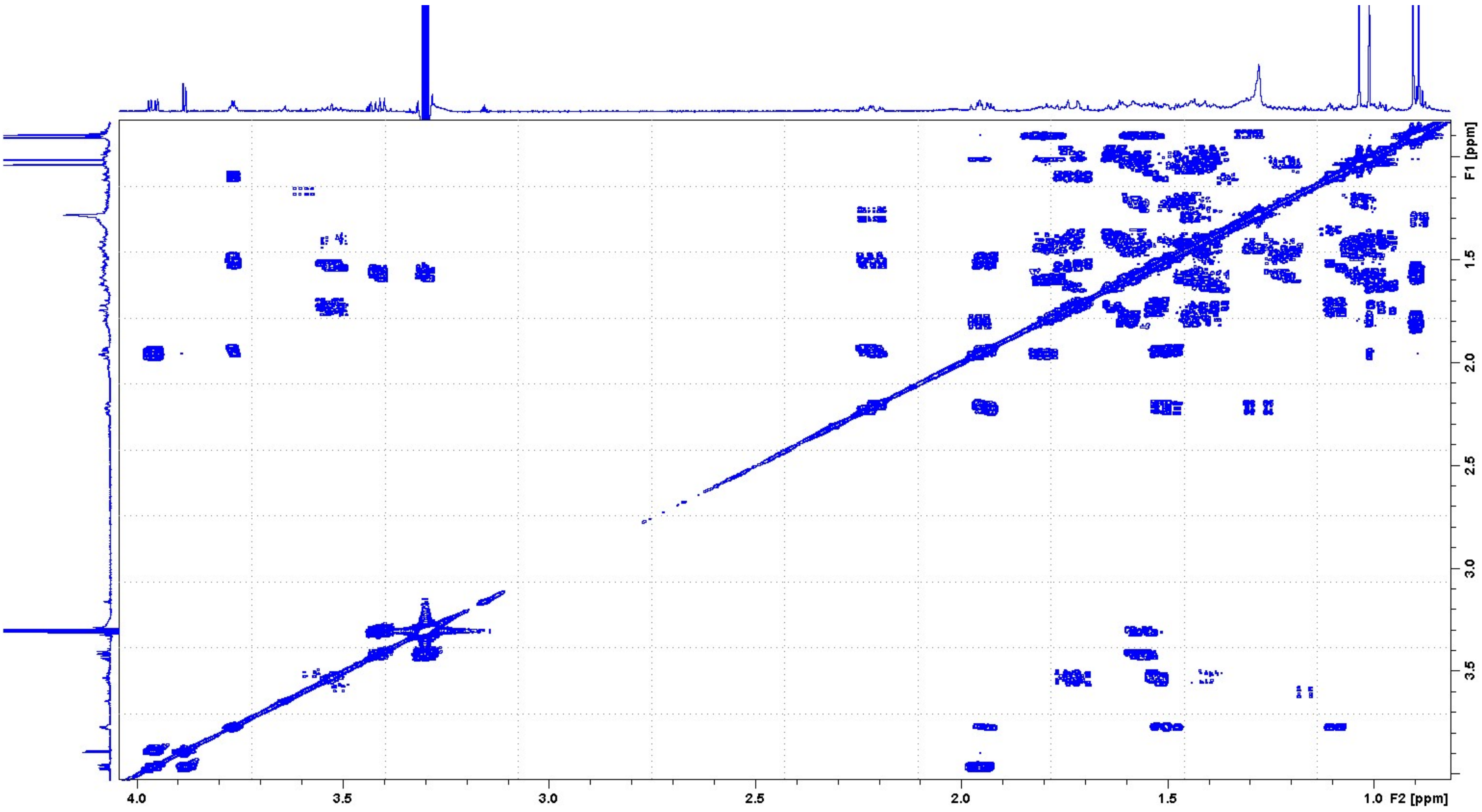

Figure S13. HSQC (700.13 MHz, CD_3OD) Spectrum of Compound **2**

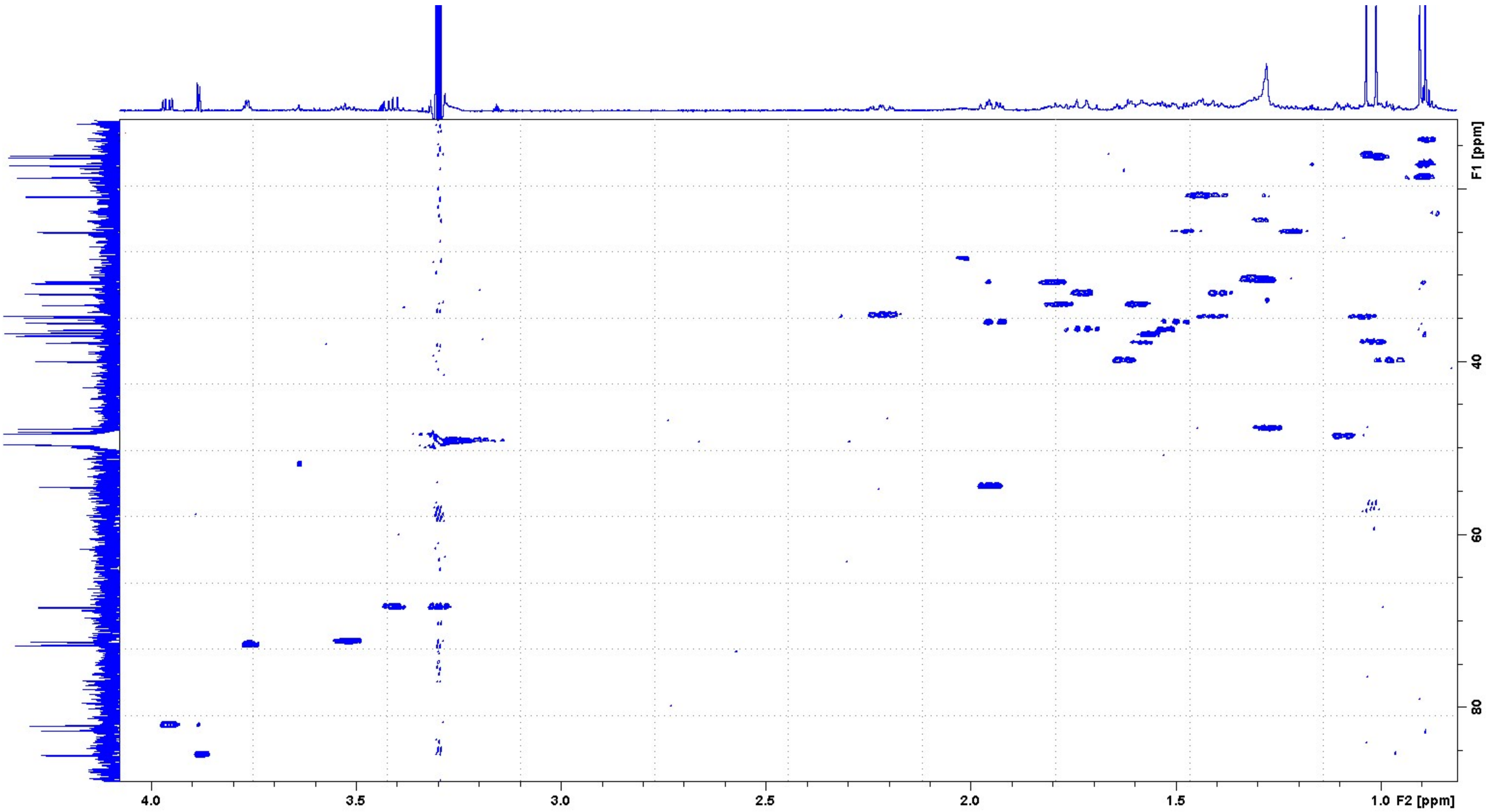

Figure S14. HMBC (700.13 MHz, CD_3OD) Spectrum of Compound **2**

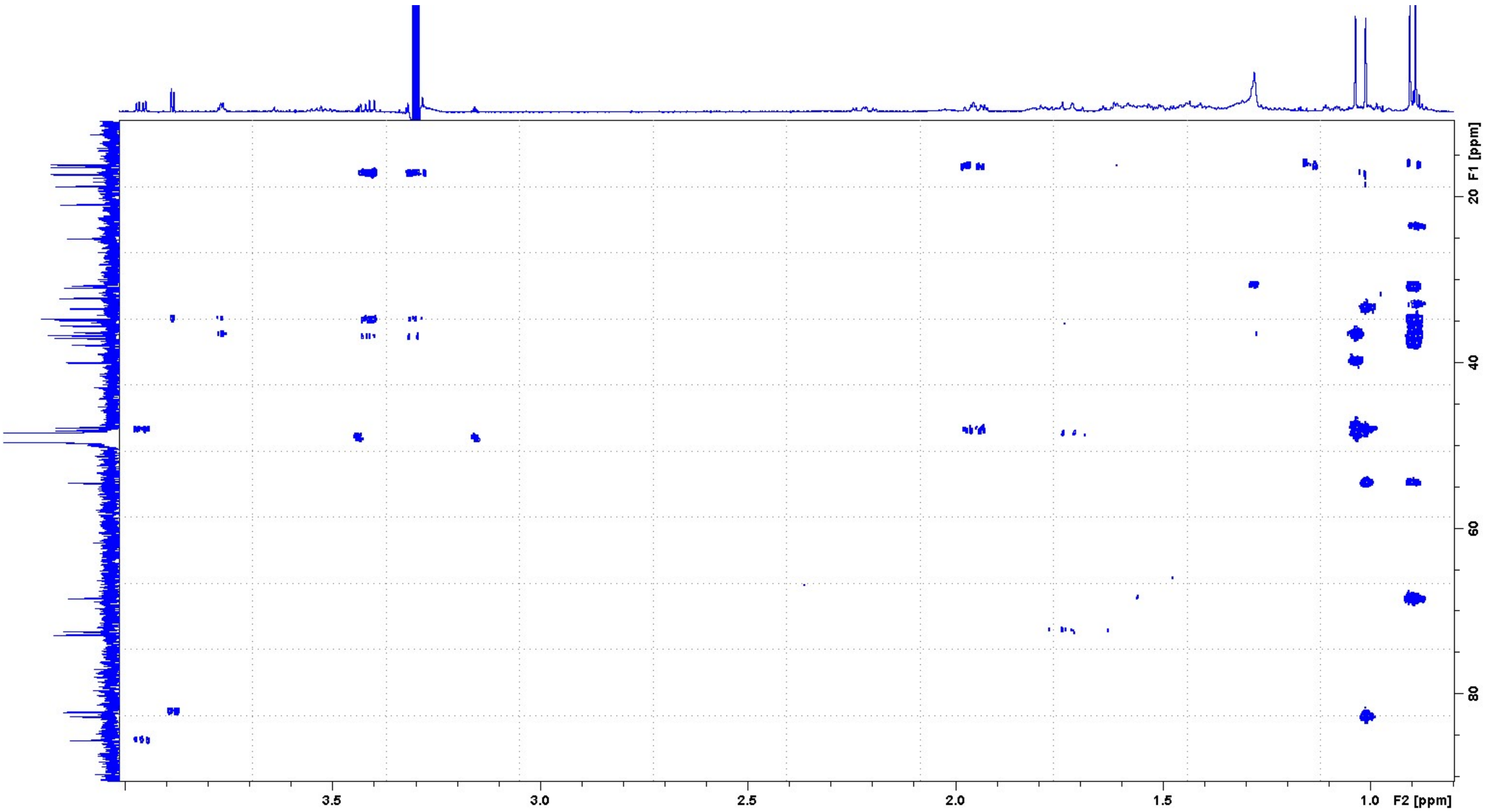

Figure S15. ROESY (700.13 MHz, CD_3OD) Spectrum of Compound **2**

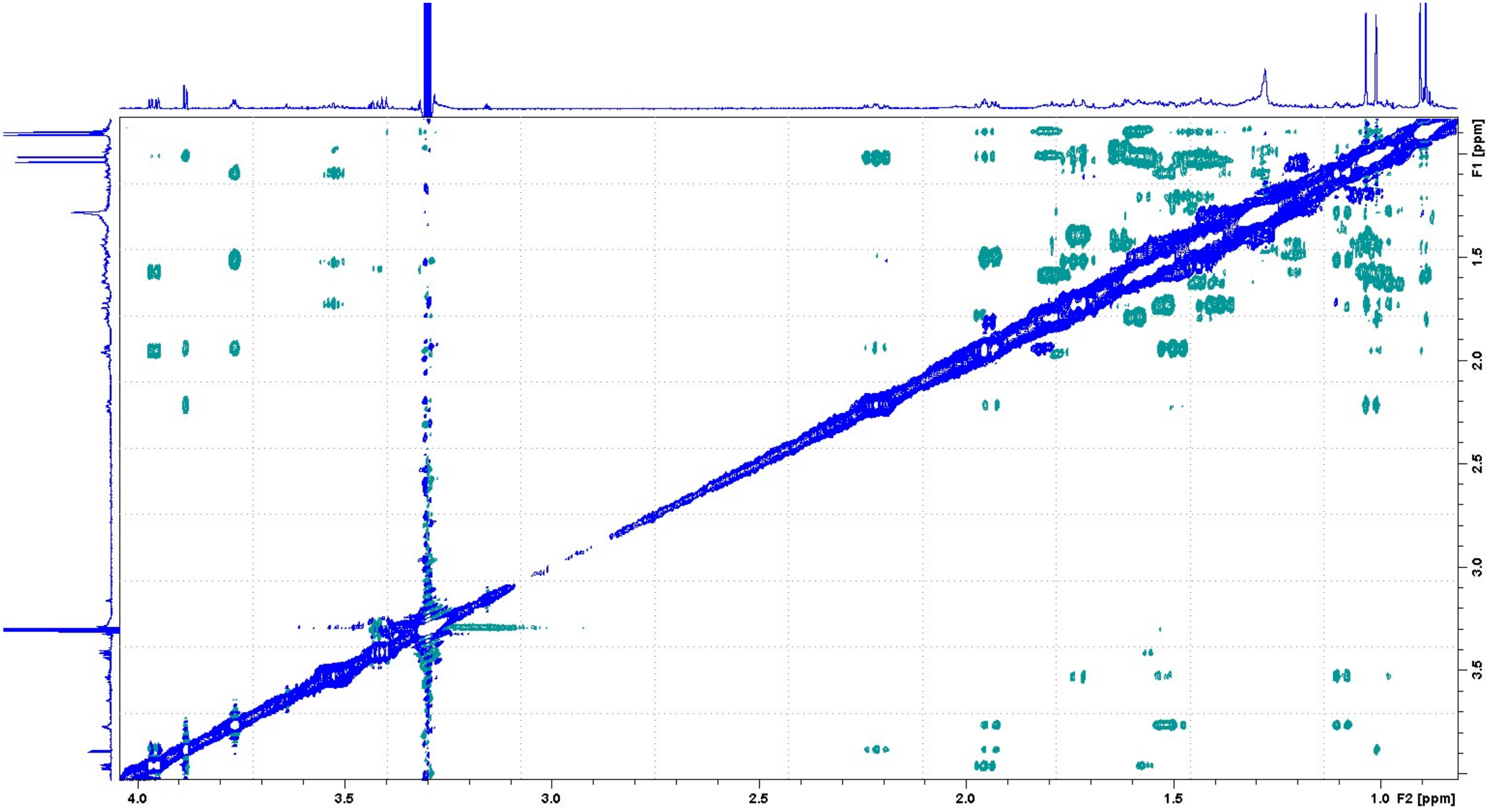

Figure S16. HRESIMS Spectrum of Compound **3**

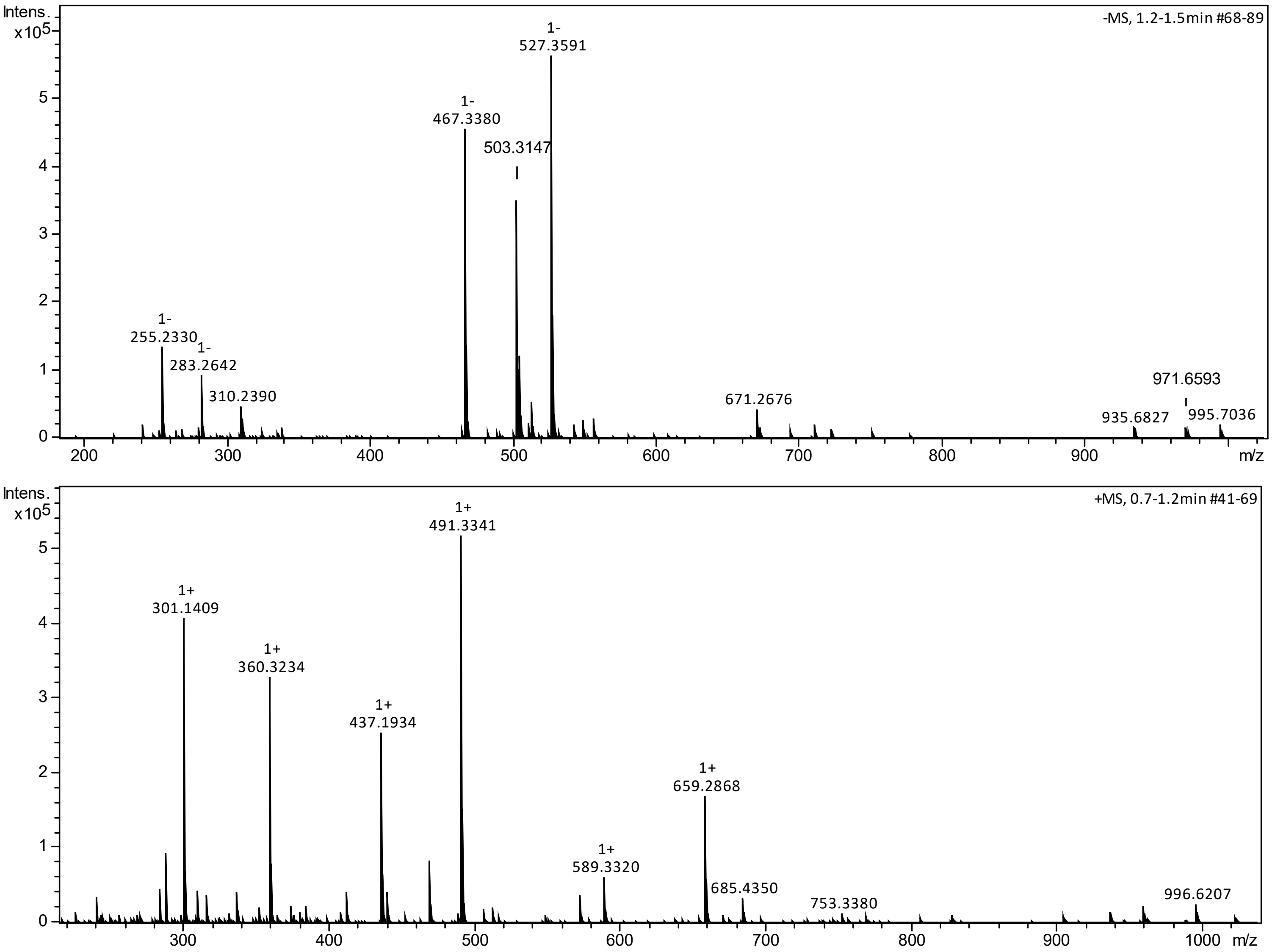

Figure S17. ^{1}H-NMR (700.13 MHz, CD_3OD) Spectrum of Compound **3**

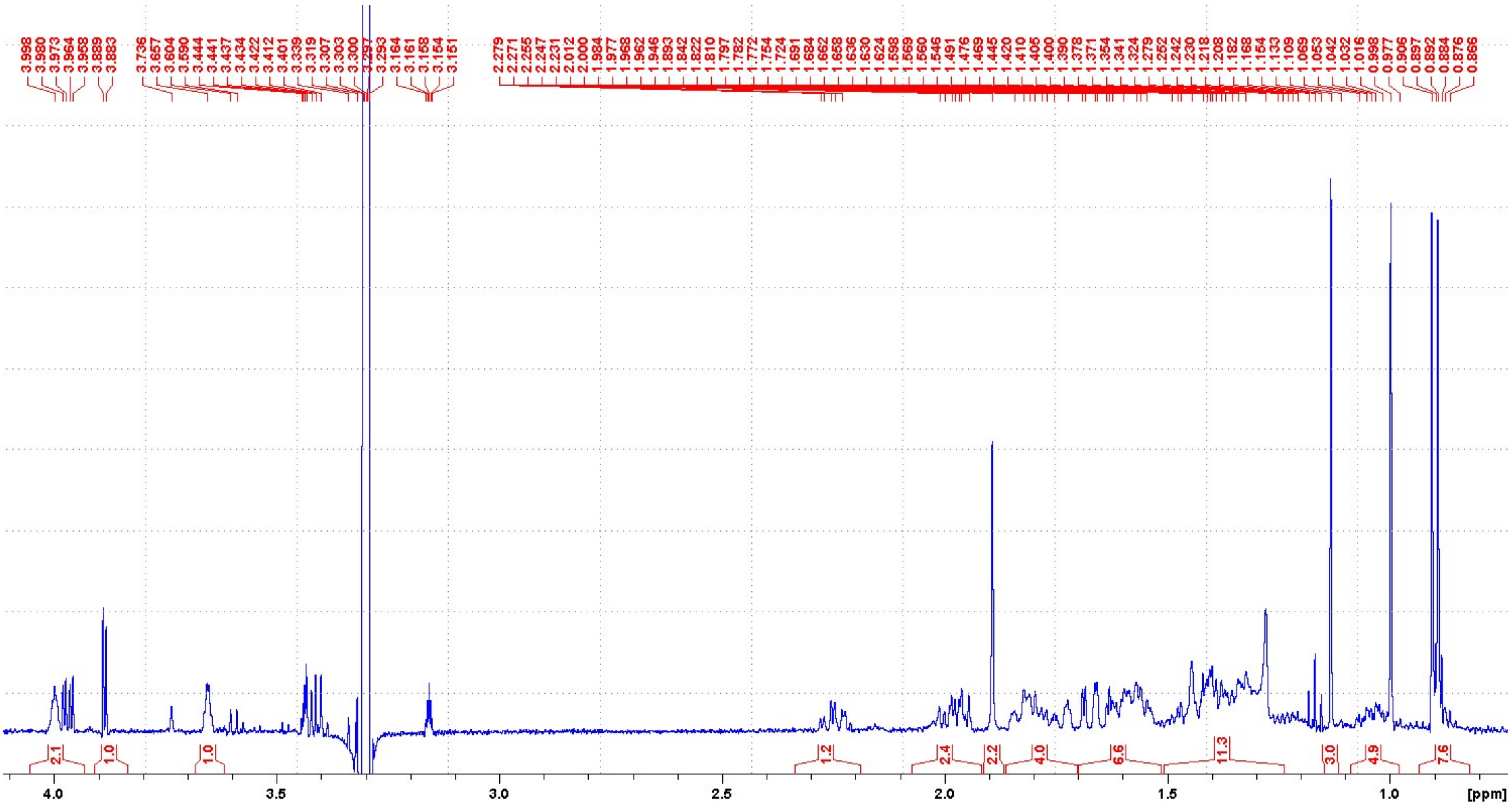

Figure S18. ^{13}C-NMR (176.07 MHz, CD_3OD) Spectrum of Compound **3**

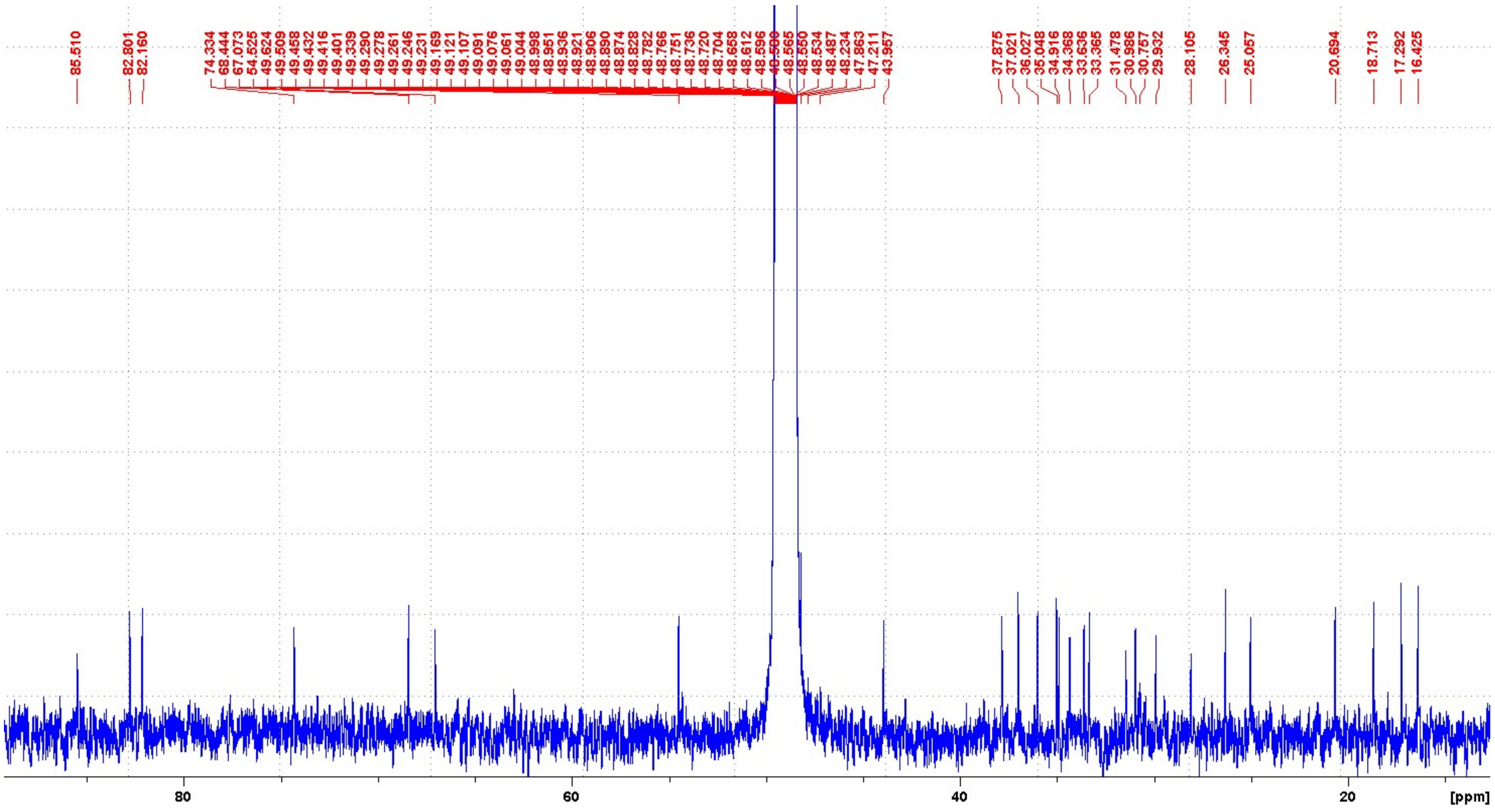

Figure S19. COSY (700.13 MHz, CD_3OD) Spectrum of Compound **3**

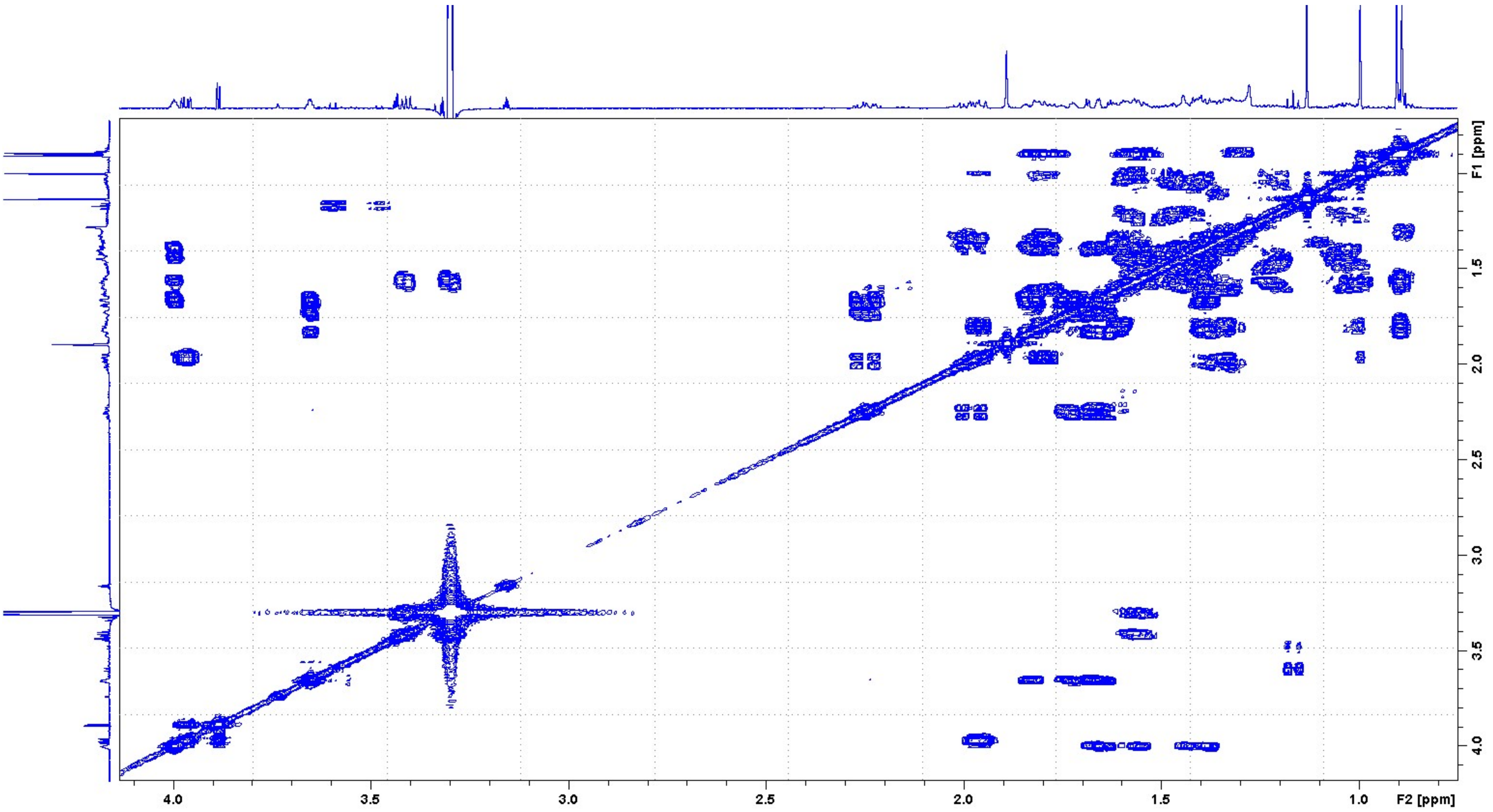

Figure S20. HSQC (700.13 MHz, CD_3OD) Spectrum of Compound **3**

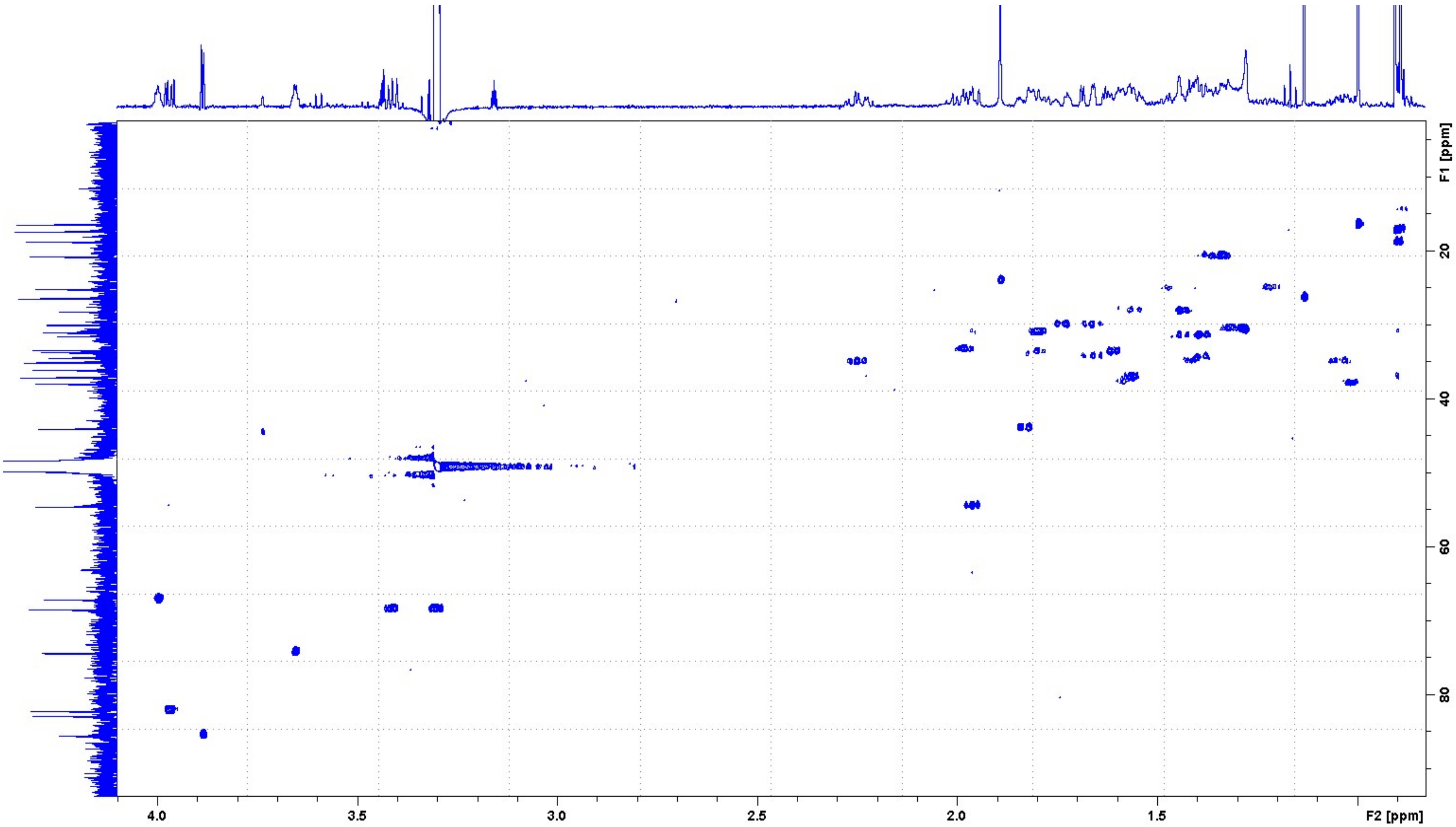

Figure S21. HMBC (700.13 MHz, CD_3OD) Spectrum of Compound **3**

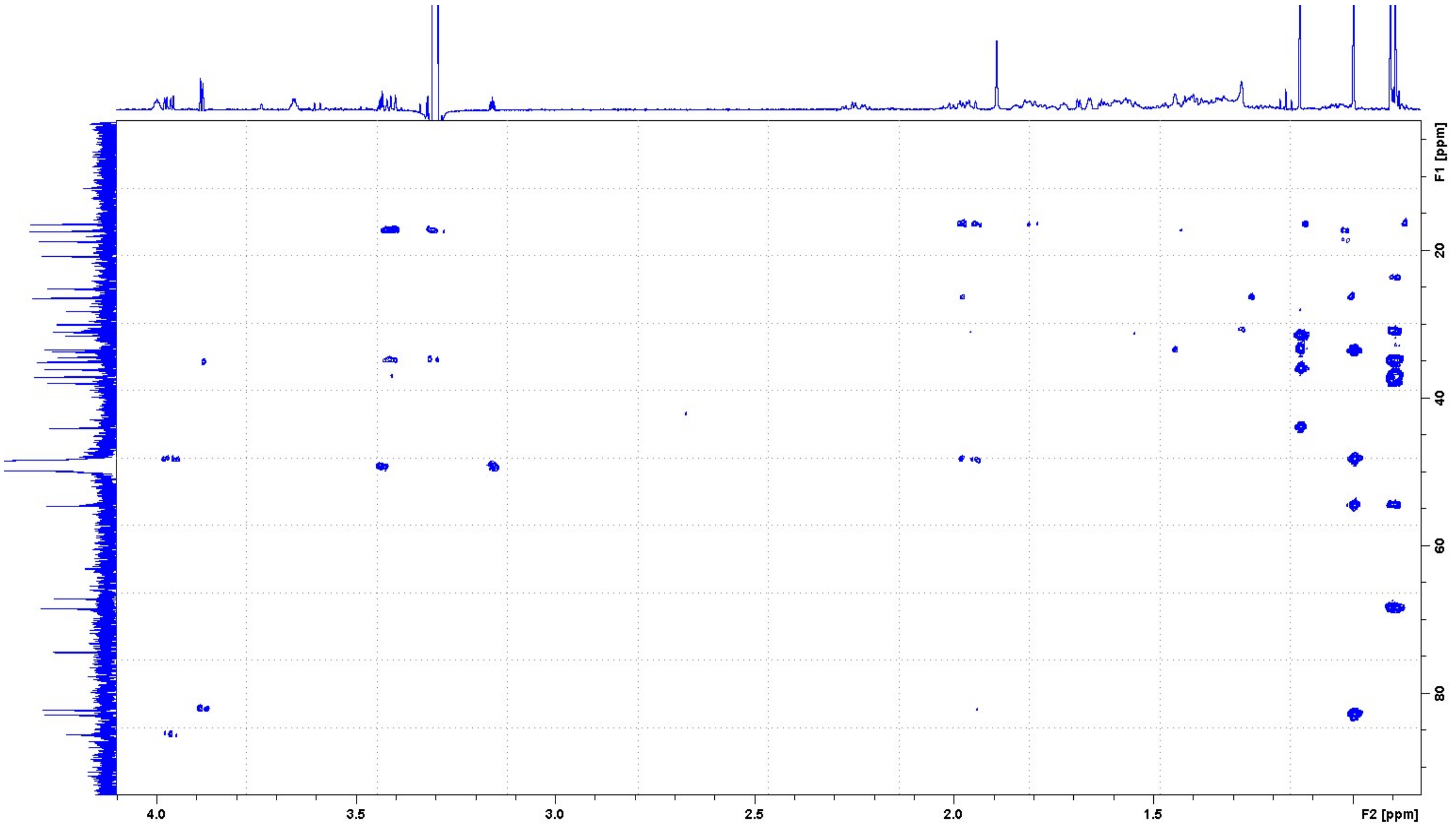

Figure S22. ROESY (700.13 MHz, CD_3OD) Spectrum of Compound **3**

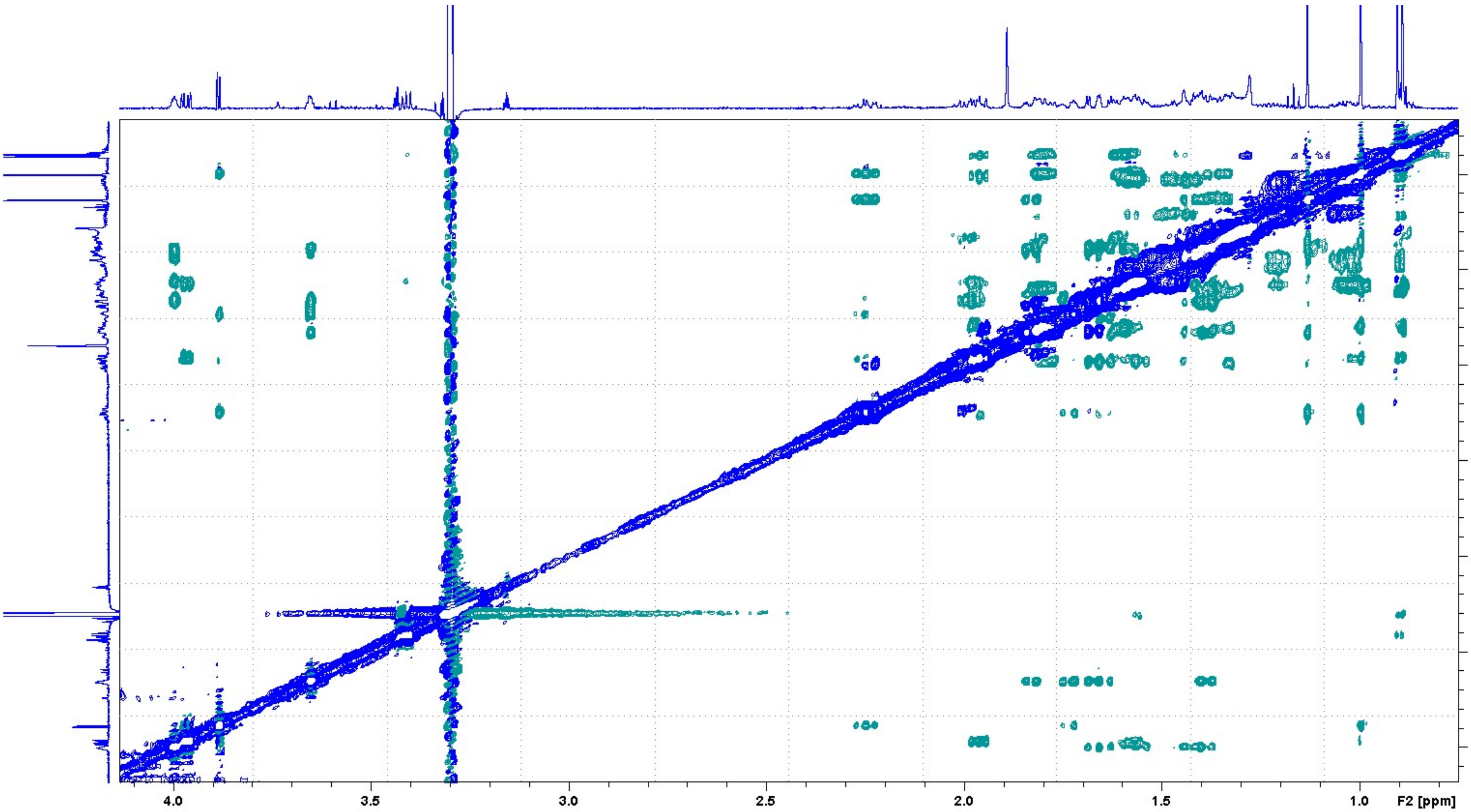

Figure S23. HRESIMS Spectrum of Compound **4**

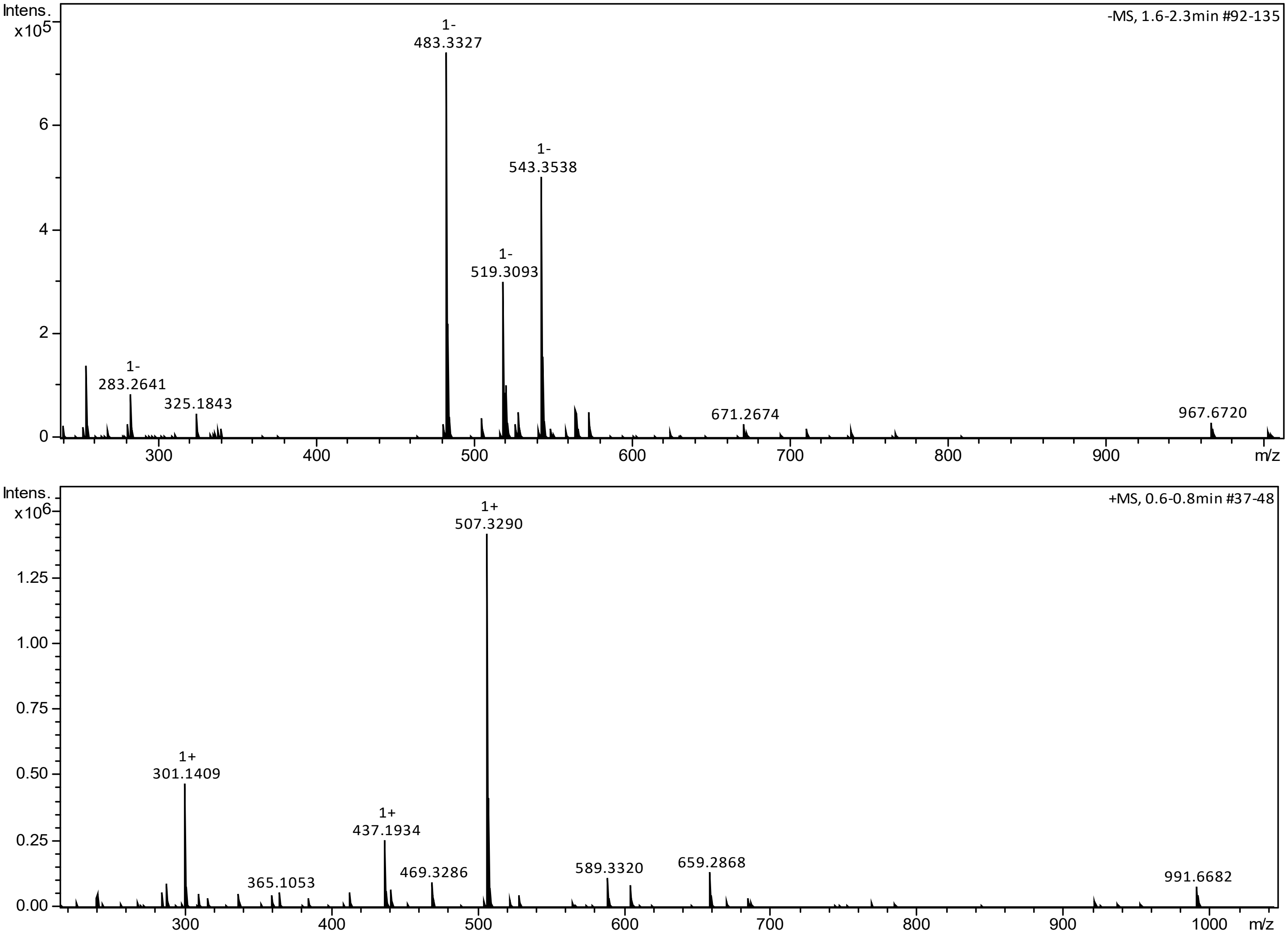

Figure S24. ^{1}H-NMR (700.13 MHz, CD_3OD) Spectrum of Compound **4**

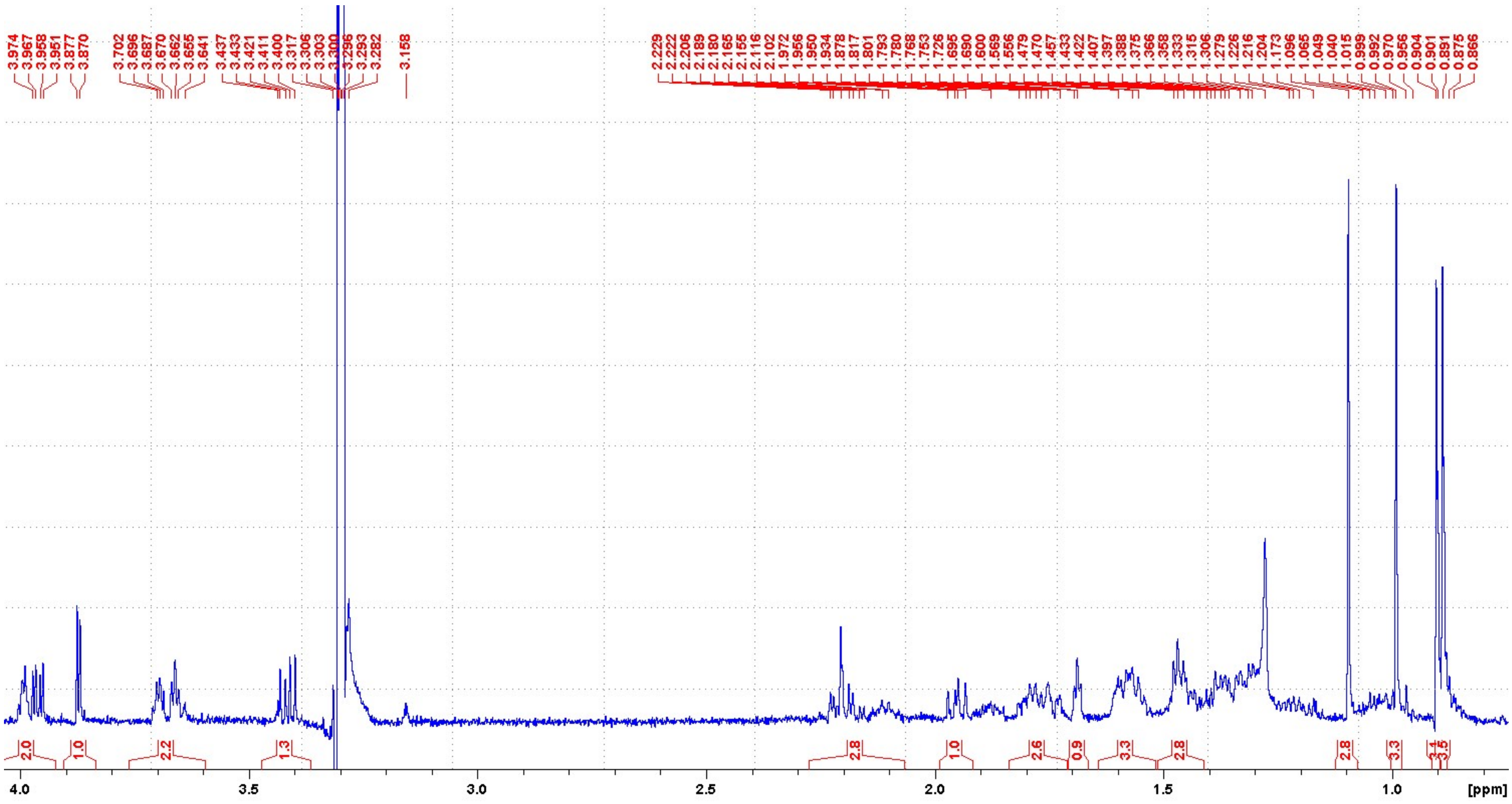

Figure S25. ^{13}C-NMR (176.07 MHz, CD_3OD) Spectrum of Compound **4**

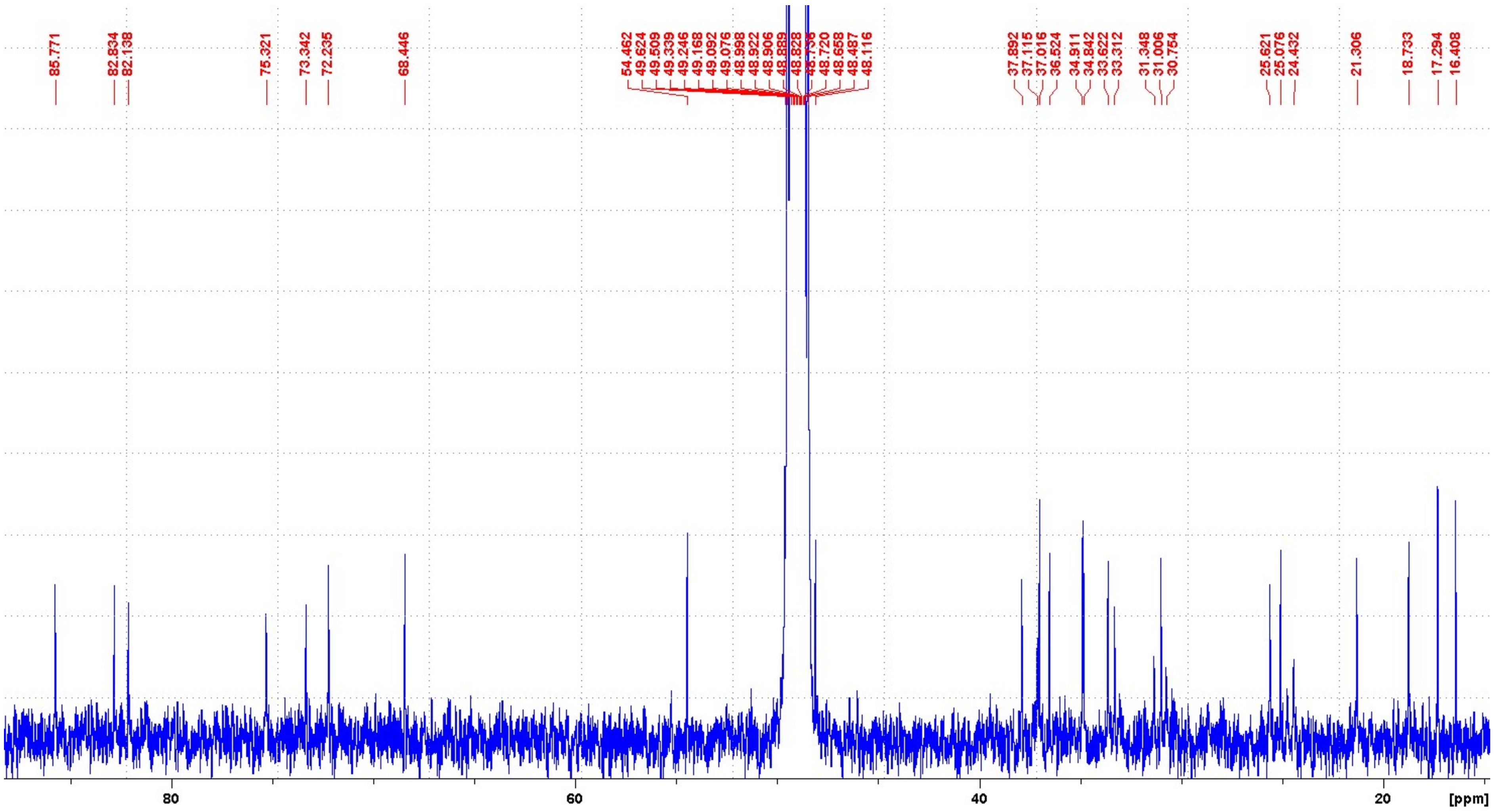

Figure S26. COSY (700.13 MHz, CD_3OD) Spectrum of Compound **4**

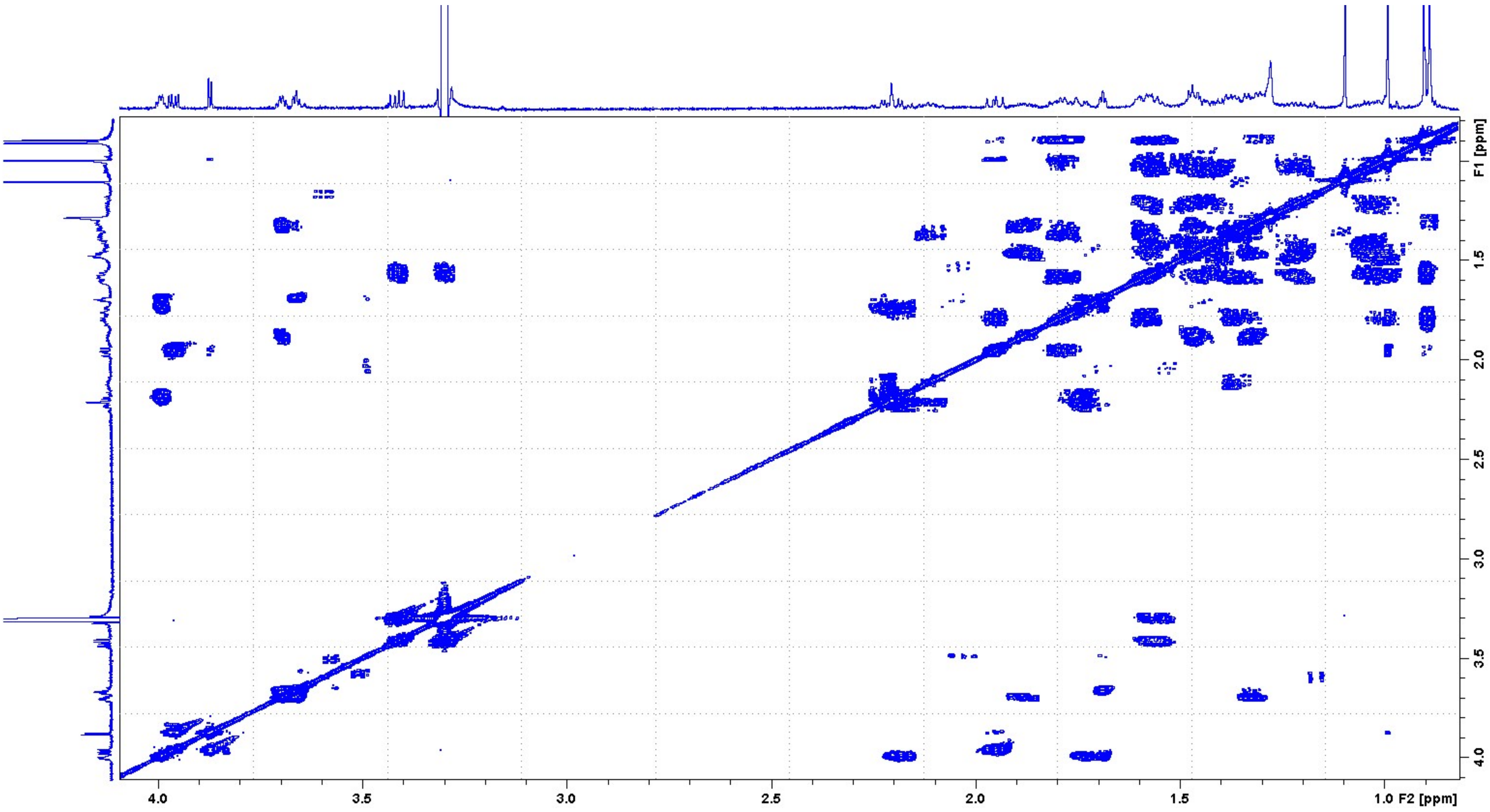

Figure S27. HSQC (700.13 MHz, CD_3OD) Spectrum of Compound **4**

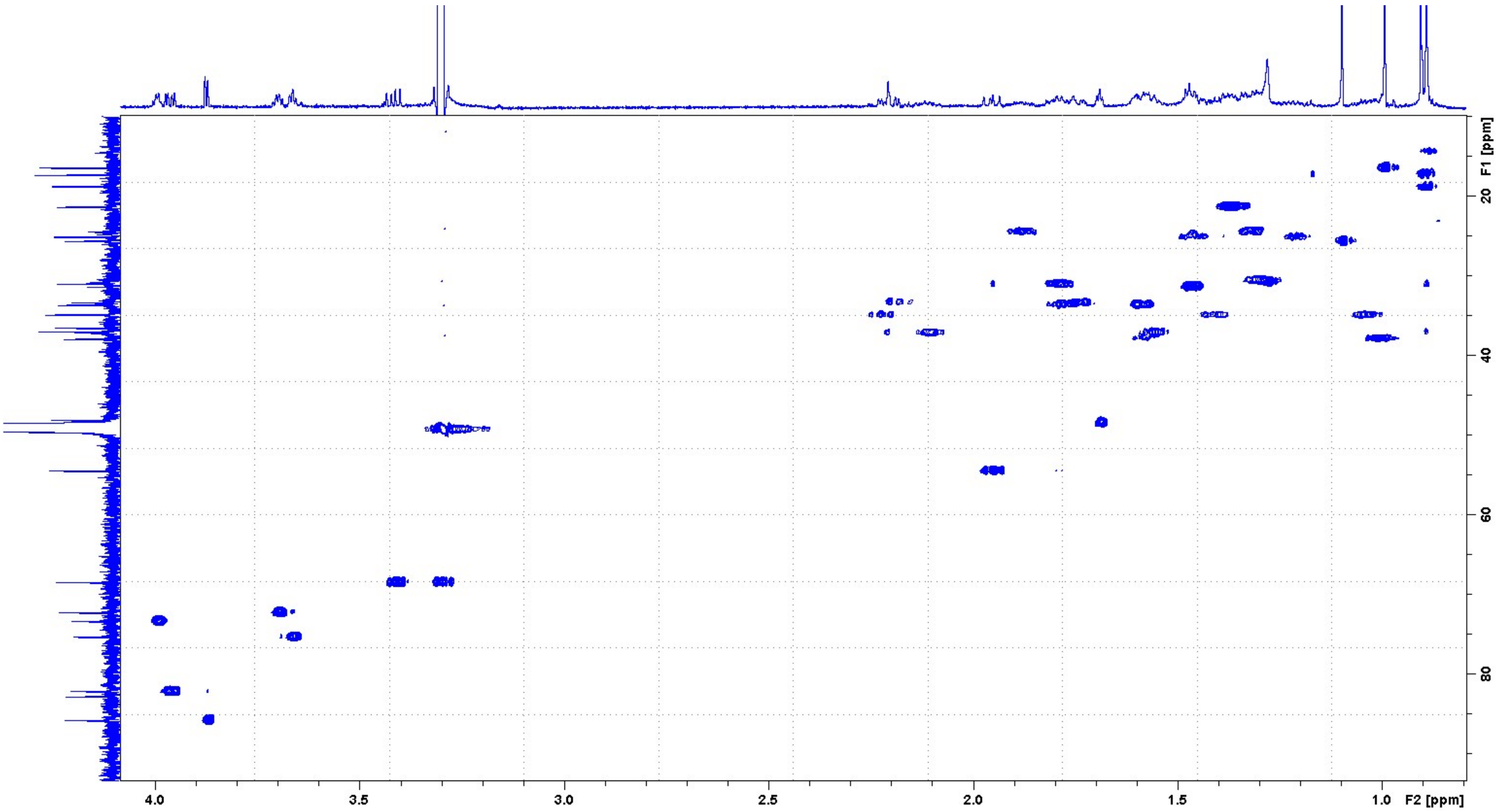

Figure S28. HMBC (700.13 MHz, CD_3OD) Spectrum of Compound **4**

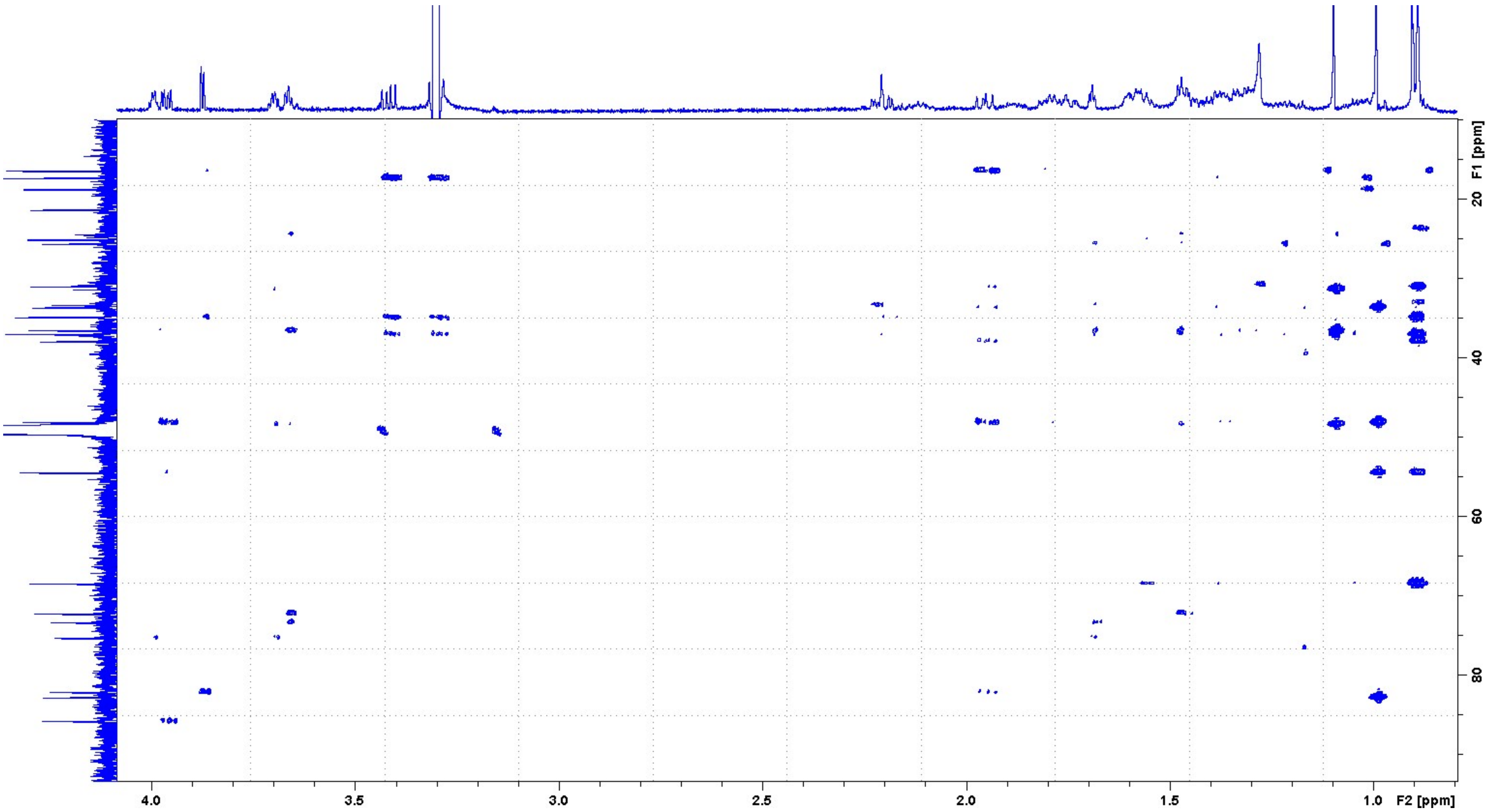

Figure S29. ROESY (700.13 MHz, CD_3OD) Spectrum of Compound **4**

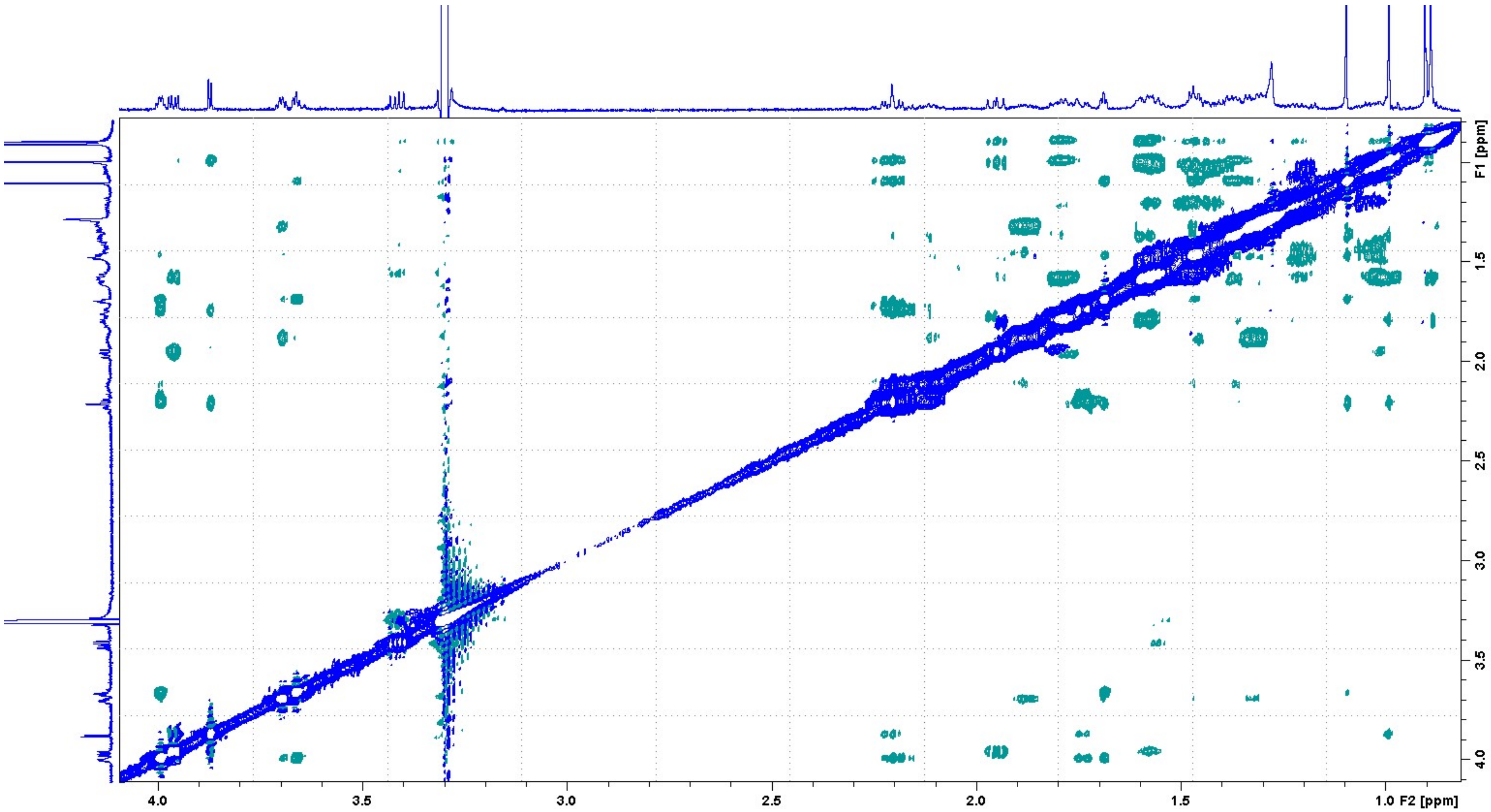

Figure S30. 1D TOCSY NMR (700.13 MHz, CD_3OD) Spectrum of Compound **4**

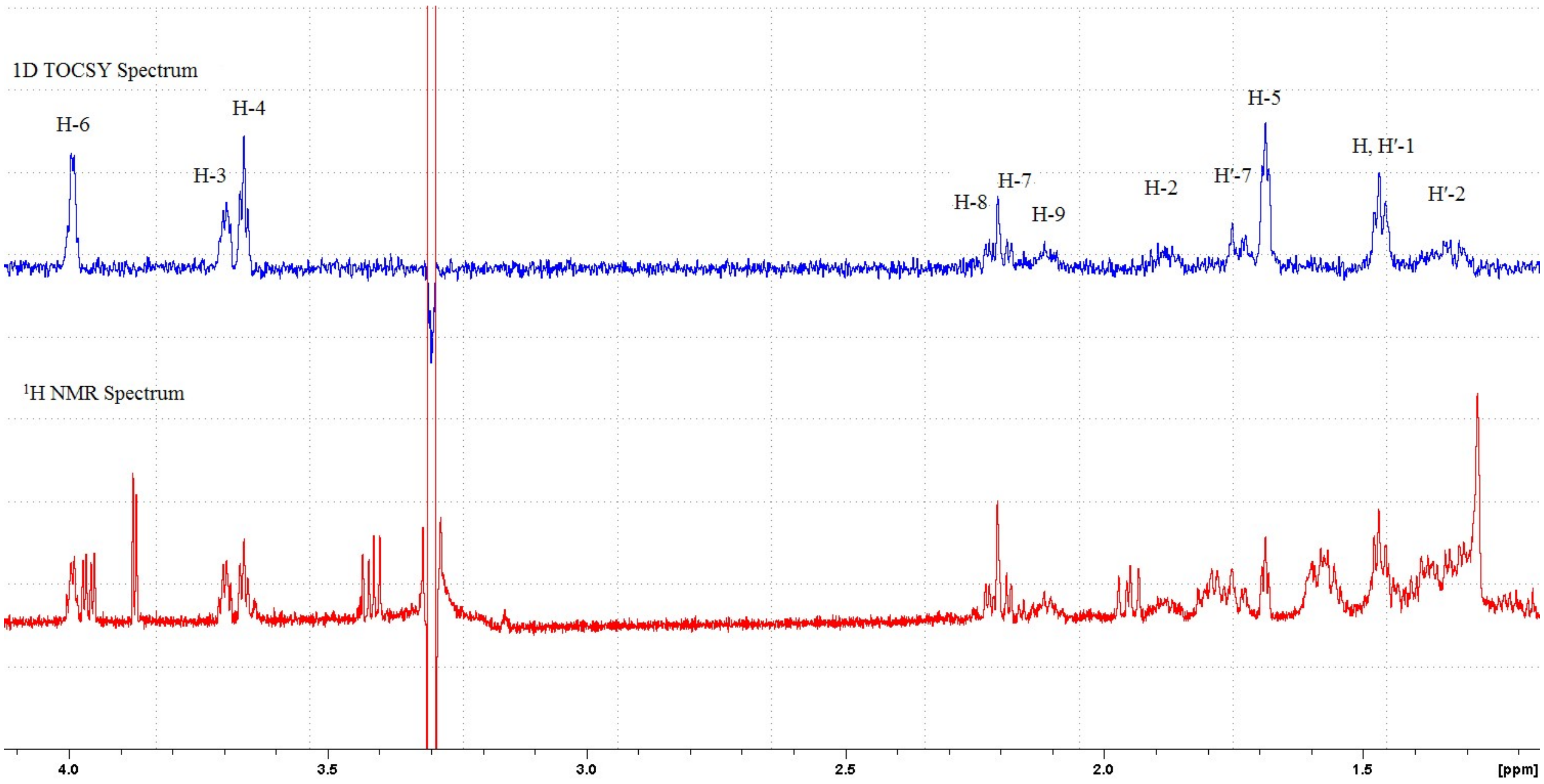

Supplement: Supplementary file 1 [file molecules-25-01440-s001.pdf]
